# A Turing Test for artificial nets devoted to vision

**DOI:** 10.3389/frai.2025.1665874

**Published:** 2026-01-05

**Authors:** Jorge Vila-Tomás, Pablo Hernández-Cámara, Qiang Li, Valero Laparra, Jesús Malo

**Affiliations:** 1 Image Processing Lab, Universitat de València, Valencia, Spain; 2 TReNDS, Georgia State, Georgia Tech, and Emory, Atlanta, GA, United States

**Keywords:** evaluation of AI models, neural networks for vision, human vision, Turing Test, low-level visual psychophysics, linear + non-linear cascade, image quality, image segmentation

## Abstract

In this work^1^ we argue that, despite recent claims about successful modeling of the visual brain using deep nets, the problem is far from being solved, particularly for low-level vision. Open issues include *where should we read from in ANNs to check behavior? What should be the read-out? Is this ad-hoc read-out considered part of the brain model or not?* In order to understand vision-ANNs, *should we use artificial psychophysics or artificial physiology?* Anyhow, *should artificial tests literally match the experiments done with humans?* These questions suggest a clear need for biologically sensible tests for deep models of the visual brain, and more generally, to understand ANNs devoted to generic vision tasks. Following our use of low-level facts from *Vision Science* in Image Processing, we present a low-level dataset compiling the basic spatio-chromatic properties that describe the adaptive bottleneck of the retina-V1 pathway and are not currently available in popular databases such as BrainScore. We propose its use for qualitative and quantitative model evaluation. As an illustration of the proposed methods, we check the behavior of three recent models with similar deep architectures: (1) A parametric model tuned via the psychophysical method of Maximum Differentiation [Malo & Simoncelli SPIE 15, Martinez et al. PLOS 18, Martinez et al. Front. Neurosci. 19], (2) A non-parametric model (the *PerceptNet*) tuned to maximize the correlation with humans on subjective image distortions [Hepburn et al. IEEE ICIP 20], and (3) A model with the same encoder as the *PerceptNet*, but tuned for image segmentation [Hernandez-Camara et al. Patt.Recogn.Lett. 23, Hernandez-Camara et al. Neurocomp. 25]. Results on the proposed 10 compelling psycho/physio visual properties show that the first (parametric) model is the one with behavior closest to humans.

## 1 Introduction

### 1.1 Prologue

This work reproduces our *talk* (otherwise unpublished in print) at the AI Evaluation Workshop in June 2022 at the AI Dept. of the University of Bristol organized by Prof. Raul Santos of the Eng. Maths Dept. of UoB (Malo et al., 2022). That *talk* proposed an original methodology (with experimental results) to evaluate deep nets devoted to vision tasks and was the seed of our current (as of 2025) work with Prof. Jeff Bowers of the Psychol. Dept. of UoB, as a low-level complement to his (high-level) proposals in Bowers et al. (2023) and Biscione et al. (2024). Journal publication of this 2022 *talk* is pertinent for a wider audience because this approach, based on low-level visual psychophysics, is still unusual in the AI and machine learning communities, despite some researchers are independently proposing very similar evaluations quite recently (Cai et al., 2025; Hammou et al., 2025). As shown below, our proposed evaluation program includes facts that go beyond the luminance, color, and contrast masking properties considered in Cai et al. (2025) and Hammou et al. (2025). The work of Rafal Mantiuk's lab shares the same spirit and focus on low-level psychophysics, but his focus on *quantitative comparison* is in contrast with our proposal, which, while including quantitative comparison, also stresses the *qualitative understanding* of the response curves. In that way, AI researchers can spot major conceptual errors in deep models easily. Moreover, as explained below, the selected visual stimuli^2^ (and associated psychophysical properties) allow us to intuitively infer modifications in the architectures in order to correct the detected errors.

### 1.2 Motivation: is that model really human-like?

The motivation for our proposal starts by reviewing the claims about how deep learning models are the ultimate tool to model the visual brain, as recalled in Bowers et al. (2023). Claims cited by Bowers et al. include Kubilius et al. (2019), Mehrer et al. (2021), Zhuang et al. (2021), Storrs et al. (2021), Rajalingham et al. (2018) and Macpherson et al. (2021), and other examples in the same vein include Cadena et al. (2019) and Burg et al. (2021). A skeptical tone about claims (Bowers et al., 2023) is a good practice in science^3^. Two examples of this skepticism regarding the eventual plausibility of models include major scientists such as *Tomaso Poggio* and *Horace Barlow*. In the 70s, Marr and Poggio proposed a taxonomy of the approaches to the vision problem: their famous *separate abstraction levels*, namely, computational, algorithmic, and implementation (Marr and Poggio, 1977; Marr, 1978). However, 42 years later, in view of the current tools to optimize models, Poggio himself questioned the separability of these levels (Poggio, 2021). This taxonomy has been inspiring for decades, but now it is under debate (Lengyel, 2024; Pillow, 2024; Malo and Hernández-Cámara, 2024; Hernández-Cámara et al., 2025c). For example, work on color illusions (Gomez-Villa et al., 2020b), on CSFs in autoencoders (Li et al. 2022), and on subjective distances between images in ANNs (Hernández-Cámara et al., 2025c; Hepburn et al., 2022) stress the relation between the computational and the algorithmic levels, thus questioning previous (purely computational) explanations that disregard architecture (Malo and Gutiérrez, 2006; Laparra et al., 2012; Laparra and Malo, 2015). In a similar vein, Horace Barlow, 50 years after his inspiring *Efficient Coding Hypothesis* (Barlow, 1959, 1961), questioned his own purely infomax approach (Barlow, 2001) ^4^.

That skepticism is the core of the spirit in Bowers et al. (2023), and also the motivation of this work, which has two key ideas:

- The use of AI techniques (e.g., deep learning) to understand the visual brain may not be as easy as people thought back in 2022, and even now. More explanatory tests are required.
- Our specific proposal here is a Turing-like test (Turing, 1950) based on 10 properties of low-level human vision (our *Decalogue*) to check if a certain artificial model behaves as the (low-level) human visual brain.

### 1.3 Structure of the paper

Section 2 states that the question *Are the models sensible from the point of view of low-level physiology and psychophysics?* remains open from the perspective of modeling and evaluation. In Section 3, we propose our contribution: an easy-to-use test (consisting of online available visual stimuli) and associated responses for qualitative and quantitative evaluation of deep learning vision models. These stimuli visually illustrate low-level phenomena described by classical *Vision Science*. In Section 4, we illustrate the proposed method through the qualitative and quantitative evaluation of three recent models: (1) a classically formulated, not end-to-end optimized model with a functional form derived from classical vision science literature, where the specific values of its parameters have been psychophysically measured (Malo and Simoncelli, 2015; Martinez et al., 2018, 2019; Malo et al., 2024). (2) A network with a bio-inspired architecture but with free parameters end-to-end optimized to reproduce subjective image quality, the *PerceptNet* (Hepburn et al., 2020). It resembles AlexNet and VGG, but it was specifically designed to accommodate the known aspects of the retina–cortex visual pathway using a constrained version of divisive normalization (Ballé et al., 2017). And (3) a model with the same encoder as the *PerceptNet*, but augmented with a decoder, and both (encoder and decoder) are trained for image segmentation (Hernández-Cámara et al., 2023; Hernández-Cámara et al., 2025b), which is also a biologically plausible task. Section 5 discusses what can be learned from the proposed test and shows an example of how a model can be fixed. Note that even in the engineering case where one does not necessarily need the networks to resemble humans, one would always want them to have good adaptation properties to achieve good generalization, and potential failures in this regard become clearly evident through the proposed tests. Finally, Section 6 concludes the paper. Supplementary materials include (a) the ground-truth values of the response curves in the proposed tests, (b) in-depth results of the CSFs of the models using six different read-out strategies at all the layers of the considered networks, and (c) a critical discussion of the aggregation of quantitative quality descriptors used in BrainScore (Schrimpf et al., 2018).

## 2 Open issues in modeling vision

As pointed out in Torralba et al. (2024), the basic question, as in human vision, is how to deal with deep models which are hardly explainable black boxes once trained.

### 2.1 Uncertain computational goal

**Figure 1:**
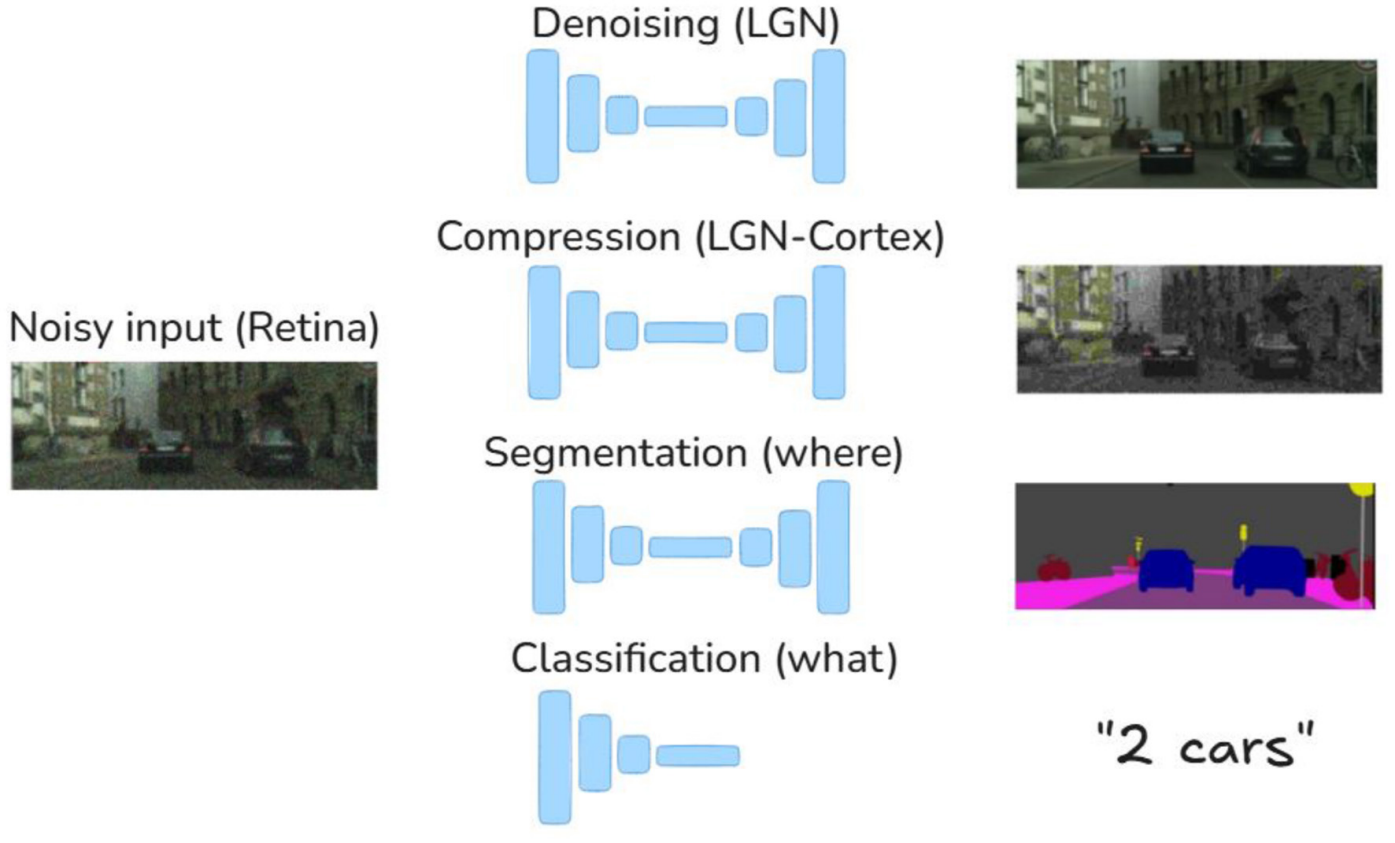
Image denoising, image compression, image segmentation, and image classification architectures with (eventually) biological correlates in the LGN, the V1, and beyond. However, it is not obvious how these tasks may be combined to explain biological vision. Images reproduced with permission from: M. Cordts, M. Omran, S. Ramos, T. Rehfeld, M. Enzweiler, R. Benenson, U. Franke, S. Roth, and B. Schiele, “The Cityscapes Dataset for Semantic Urban Scene Understanding,” in *Proc. of the IEEE Conference on Computer Vision and Pattern Recognition (CVPR)*, 2016.

First, the more general open issue is the discussion on the *computational goal* that eventually explains the organization and behavior of visual systems. Consider architectures/tasks such as the ones presented in Figure 1. These tasks are related to low-, mid-, and high-level tasks arguably implemented by biological vision. In biology, enhancement of the blurry and noisy signal in the retina has been proposed as an explanation of the LGN, as pursuing this goal may reproduce some of its spatio-chromatic (Atick et al., 1992; Li et al., 2022) and purely chromatic (Gomez-Villa et al., 2020b) features. Another example is the compression, possibly, happening in part at the LGN bottleneck and at the feature selection after V1. Bandwidth limitation, dimensionality reduction, and attention focus are sensible goals in this regard (Karklin and Simoncelli, 2011; Lindsey et al., 2019; Zhaoping, 2014). A number of compression algorithms [for images (Wallace, 1991; Malo et al., 1995, 2000a; Taubman and Marcellin, 2013; Malo et al., 2006; Ballé et al., 2017) and video Le Gall, 1992; Malo et al., 2000b,c, 2001] have been based on human vision models. Segmentation is arguably another (mid-level) task that has to be done by biological vision, and biological non-linearities have been shown to improve segmentation in images (Hernández-Cámara et al., 2023; Hernández-Cámara et al., 2025b) and video (Malo et al., 2000c, 2001). Arguably, segmentation is implemented in the *where* channel from the lower-level primitives extracted in V1 (Goodale et al., 1991; Milner and Goodale, 1992). Higher-level tasks such as classification are supposed to happen in the *what* channel (Logothetis and Sheinberg, 1996; Kreiman et al., 2000). Similarly, in standard models such as the one depicted in Figure 1, biological non-linearities have been shown to have a significant role in classification (Coen-Cagli and Schwartz, 2013; Miller et al., 2022).

### 2.2 Uncertain read-out mechanisms

**Figure 2:**
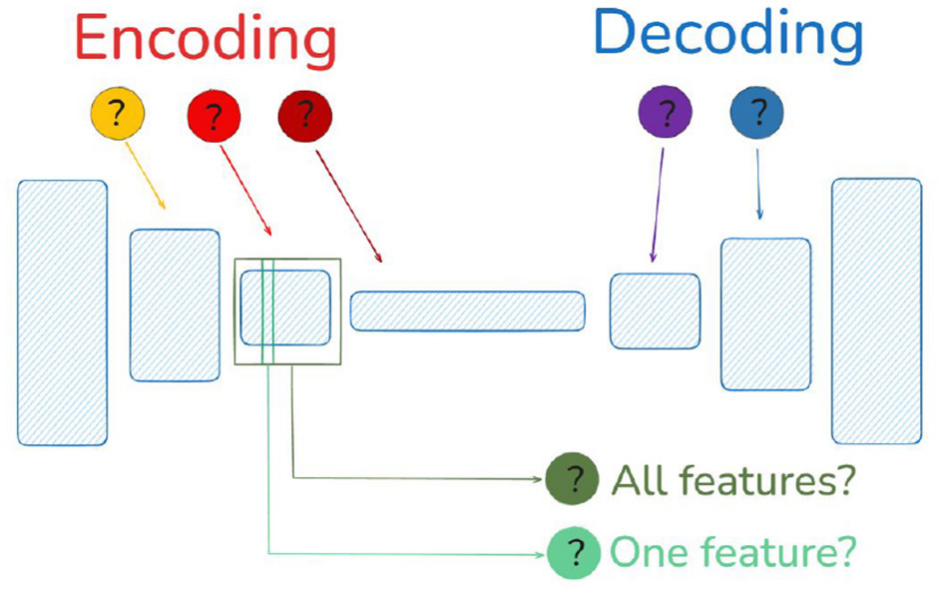
Given a deep model successfully trained for some visual task, the read-out location and read-out mechanism (or decoder) are important to assess its biological plausibility.

As stated in the introduction, in the age of automatic differentiation where the classical Marr-Poggio levels are not that separated, the *computational goal* is not the only open issue. For instance, in order to check if a (mathematical) model is biologically sensible, where should we read the signals from? The read-out mechanism is also important. Note that the fact that a certain layer has the necessary information in order to solve a task (read-out in *any complicated* way, e.g., a highly specialized dense network) is not enough to say that this layer represents the way the visual brain works: the necessary information is already present in the retina (if read in a proper way) and, of course, the retina is not a good model for the rest of the visual brain. This problem is illustrated in Figure 2.

**Figure 3:**
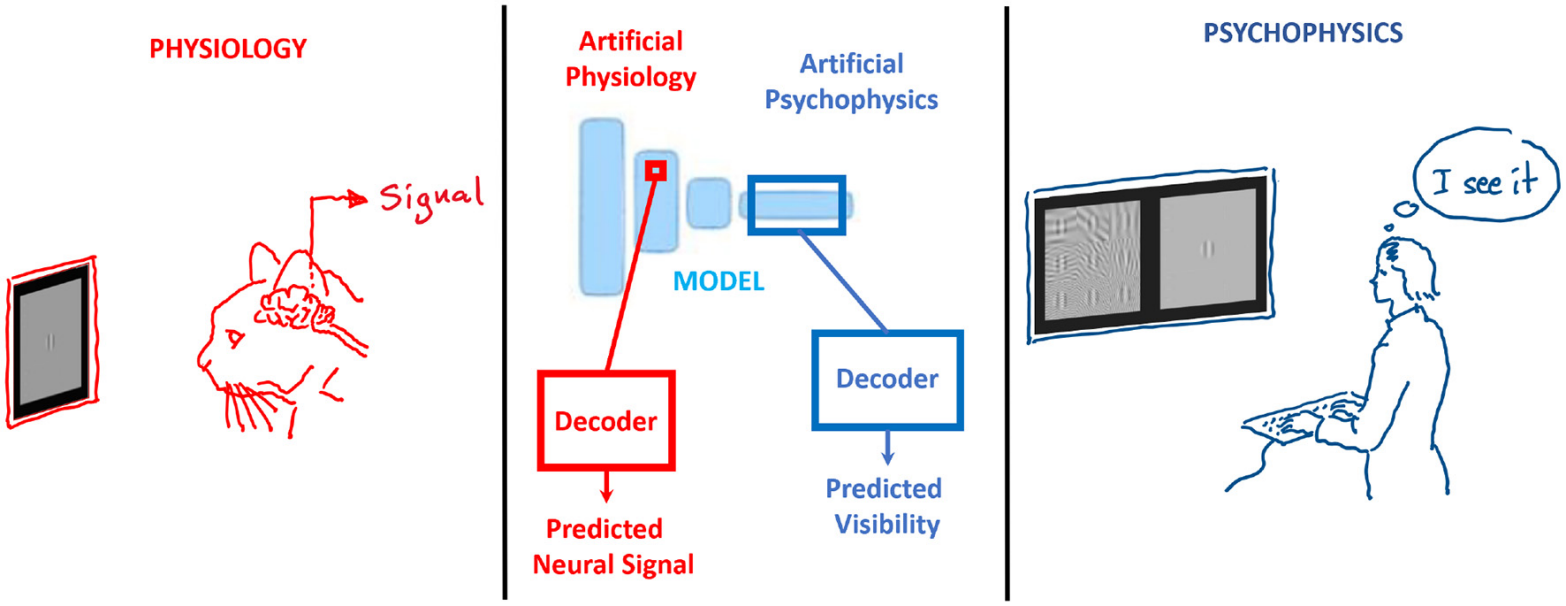
In artificial physiology **(Left)** and in artificial psychophysics **(Right)**, the arbitrary decoder to read out model activations is critical.

In the case of doing *artificial physiology*, i.e., reading the signals from certain neurons or layers, or *artificial psychophysics*, i.e., trying to make decisions from the responses of the network to decide if a certain stimulus is visible or not, one should propose a *read-out mechanism* to summarize the responses into a decision variable (see Figure 3). The selection of the *read-out mechanism* is not trivial. In fact, the quality of the read-out information may strongly depend on the complexity of this (arbitrarily selected) mechanism. As a result, one may not be able to tell if the model itself is good, or if the good behavior has to be attributed to a clever read-out that is not part of the model. Examples include the use of classifiers at certain locations of the network to make a decision on visibility, as in (Coen-Cagli and Schwartz, 2013; Akbarinia et al., 2023), or without classifiers relying on the model output (Hernández-Cámara et al., 2025a); or the (more classical) use of Euclidean distances between stimuli to tell if they are discriminable (Teo and Heeger, 1994; Li et al., 2022; Hernández-Cámara et al., 2025c). These (arbitrary) decisions definitely affect the characterization of the system, e.g., its frequency response (Li et al., 2022; Akbarinia et al., 2023). For example, linear or non-linear classifiers effectively apply different (non-Euclidean) distance metrics (Duda and Hart, 1973) and, hence, they should lead to different decisions.

Another (more particular) discussion is the debate on the summation, which is classical in vision science (Graham, 1989): for instance, which Minkowski exponent is more physiologically plausible? Note that using different norms and summation schemes definitely leads to different results (Laparra et al., 2010). A final (also non-obvious) way of assessing stimuli in the network is measuring differences in the statistical properties of the response (Wang et al., 2004; Ding et al., 2022) or measuring information flow along the network (Sheikh et al., 2005; Sheikh and Bovik, 2006; Malo, 2020; Malo et al., 2021; Li et al., 2024). These options require making non-trivial decisions such as which statistical descriptors make sense (Gatys et al., 2016; Ding et al., 2022), or how to set the level of noise in the network (Sheikh et al., 2005; Sheikh and Bovik, 2006; Malo, 2020). In this regard, models can be improved either by changing the architecture and the measures of information (Malo et al., 2021; Laparra et al., 2025), or by better estimations of the internal noise (Malo et al., 2025).

### 2.3 Uncertain experimental setting

**Figure 4:**
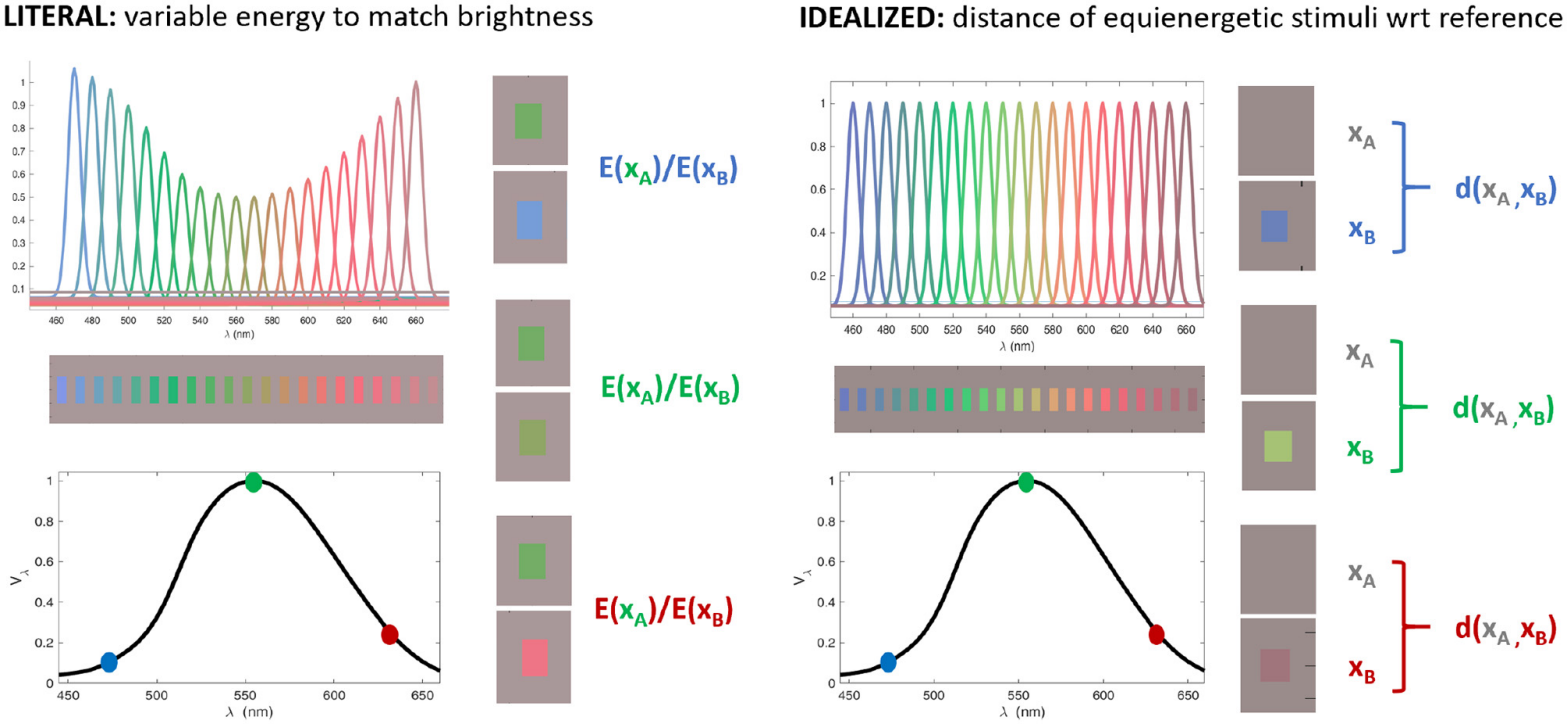
In measuring the spectral sensitivity of certain elements of a network, one may try a *literal* reproduction of human psychophysics **(Left)** or an *idealized* experiment **(Right)**. The *literal reproduction* could be done through matching experiments (Wyszecki and Stiles, 2000): finding the ratio of energies necessary to match the response to quasi-spectral stimuli of different wavelengths. The *idealized* version of the experiment could be based on measuring the increment of response (distance) due to equal energy quasi-monochromatic stimuli with regard to a common reference.

And finally, the third open issue is the way of doing the evaluation: *the experiment implementation matters*. In particular, *should we use artificial physiology or artificial psychophysics?* Current techniques by the machine learning community to visualize the behavior of the networks (Mahendran and Vedaldi, 2016; Luo et al., 2016) are based on classical single-cell recordings, such as the very concept of *receptive field* (Hubel et al., 1959; Hubel and Wiesel, 1961; Ringach, 2002), and the identification of sensitive neurons by looking at the stimulus that maximizes the neuron response, which is a common practice in visual neuroscience (Tailby et al., 2008). However, there are more sophisticated techniques such as *reverse correlation* which are used both in physiology (Ringach and Shapley, 2004) and in psychophysics (Eckstein and Ahumada, 2002), and these are not yet widely used in machine learning. Regarding the experimental setting, *should one go for a literal reproduction of the experiments with humans, or should one try an idealized version of the experiment?* This open question can be illustrated by the example in Figure 4 on the spectral sensitivity of a network.

This is a non-trivial question because, for instance, some techniques to assess visual illusions in a model involves the inversion of the inner representation (Otazu et al., 2010; Gomez-Villa et al., 2020b), which *does not happen in the human brain*, while others, similarly to human psychophysics (Ware and Cowan, 1982), are based on *matching* the response at the inner representation (Gomez-Villa et al., 2020b, 2025). As stated above, this has implications for deciding at which layer one should impose the matching (or where to read from).

### 2.4 Better evaluation techniques are needed

All these non-trivial decisions (despite that they all belong to low-level characterizations of the visual system) clearly point out the need for better methodologies for model evaluation in order to assess how close different models may be to the visual brain. These better methods should easily show the impact of the open issues mentioned above.

In this context, our proposal here is simple: *just provide the code to generate a set of well-selected stimuli that illustrate a number of classical low-level visual psychophysics facts and have them prepared as inputs to evaluate image-computable models*. The first version of such a *low-level Turing Test* (back in 2022) included stimuli for 10 wellknown behaviors (our *Decalogue*). That Decalogue is being extended to 20 properties in our on-going (2025) collaboration with Prof. Bowers (Malo and Bowers, 2024).

The selected stimuli here (which include color and texture) are behind the current understanding of early vision as a set of linear-non-linear layers (Rust and Movshon, 2005; Schütt and Wichmann, 2017; Martinez et al., 2018, 2019; Bertalmío et al., 2020; Malo et al., 2024; Bertalmío et al., 2024). Our proposal follows the tradition of previous (too simple) low-level datasets such as the OSA ModelFest initiative (Carney et al., 1999), but low-level psychophysics has not been extensively included in the (today's popular) BrainScore (Schrimpf et al., 2018), nor in the high-level criticisms made by Bowers et al. (2023) and Biscione et al. (2024).

## 3 Our proposal: a low-level vision Turing Test for deep-nets

### 3.1 The Decalogue: facts and foundations

The set of facts and associated stimuli included in our proposal is summarized in Table 1. Among the rich literature on low-level visual psychophysics, the selection of those specific properties is grounded in two main reasons.

**Table 1:** Properties of human vision (and associated stimuli) of our Decalogue that are behind the current understanding of the information bottleneck happening between the retina and the V1 cortex.

	**Facts / properties**	**Stimuli**	**Modality**	**Response**
1	Spectral sensitivities (achromatic and opponent)	Quasi-spectral	Color	Linear
2	Brightness & color response saturation	Color calibrated	Color	Non-linear
3	Achromatic contrast sensitivity (bandwidth)	Achrom. Gabors/noise	Texture	Linear
4	Chromatic contrast sensitivity (bandwidth)	Chrom. Gabors/noise	Texture	Linear
5	Spatio-chromatic receptive fields	Deltas / noise	Texture	Linear
6	Non-linear contrast response: saturation	Gabors/noise	Texture	Non-linear
7	Non-linear contrast response: freq. dependent	Gabors/noise	Texture	Non-linear
8	Context effects: energy	Gabors/noise	Texture	Non-linear
9	Context effects: frequency	Gabors/noise	Texture	Non-linear
10	Context effects: Orientation	Gabors/noise	Texture	Non-linear

They include color, texture, and motion processing abilities of human early vision. In the original literature, the stimuli were specifically designed to probe the linear or the non-linear behavior of the system.

**First**, they describe the visual information adaptively captured (and discarded) by the front end of human vision. On the one hand, linear sensitivities describe the spectral, chromatic, and spatio-temporal bandwidth and relative weight given by the system to the frequency components of the input stimuli. This linear description in terms of sensitivity filters is the first-order approximation to the visual bottleneck. More interestingly, this bottleneck is adaptive: in classical models of vision science, extra non-linear mechanisms are proposed between the linear filters to account for the adaptive responses to the specific eigen-stimuli of the linear filters. The stimuli in the tests we compile here were specifically designed to probe those linear and non-linear mechanisms of human vision. The power and relevance of the selected stimuli for a complete characterization of the low-level bottleneck of image-computable models is suggested by the fact that, for decades, the straightforward use of these facts (with minor or no optimization at all) led to competitive image (Wallace, 1991; Malo et al., 1995, 2000a; Taubman and Marcellin, 2013; Malo et al., 2006) and video coding algorithms (Le Gall, 1992; Malo et al., 2000b,c, 2001) and distortion metrics (Daly, 1993; Null, 1993; Teo and Heeger, 1994; Malo et al., 1997; Watson and Malo, 2002; Laparra et al., 2010) equipped with color constancy and contrast adaptation (Hernández-Cámara et al., 2023; Hernández-Cámara et al., 2025b; Hernández-Cámara et al., 2024). Checking if the response of a network is human-like for those stimuli would imply that the bottleneck of the network would have *statistically* good adaptive behavior (Olshausen and Field, 1996; Schwartz and Simoncelli, 2001; Malo and Gutiérrez, 2006; Malo and Laparra, 2010; Laparra et al., 2012; Laparra and Malo, 2015; Gomez-Villa et al., 2020a; Malo, 2020, 2022). This adaptivity could be useful for the mentioned applications and also for domain adaptation (Sengar et al., 2025).

**Second**, effects elicited by the selected stimuli are visually compelling and hence, the user of the test can check (by the eye) if the model under consideration behaves like humans or not. On the one hand, sensitivity surfaces to simple (isolated) stimuli are standardized and ready for direct quantitative comparison (Wyszecki and Stiles, 2000; Hurvich and Jameson, 1957; Krauskopf and Gegenfurtner, 1992; Campbell and Robson, 1968; Mullen, 1985; Georgeson and Sullivan, 1975; Daly, 1990; Malo et al., 1997; Kelly, 1979; Díez-Ajenjo et al., 2011). On the other hand, as illustrated below, non-linear responses when using stimuli in a context (under adaptation) have specific qualitative behaviors that are easy to see (Foley, 1994; Watson and Solomon, 1997; Martinez-Uriegas, 1997). In this way, that eventual model deviations from human-like behavior are easy to detect. Moreover, Section 3.3 proposes ways to summarize these visual behaviors in a numerical score. The proposed tests do not give definitive answers to the points raised in Section 2, but they are useful to stress the impact of those issues in easy-to-view ways and rule out models accordingly.

### 3.2 The Decalogue: specific examples

In this section, we show four examples of the proposed Decalogue with series of calibrated stimuli (from the colorimetric and the spatial perspectives) that illustrate the non-linear response of humans to (i) luminance in different backgrounds leading to different perceptions of *brightness*, (ii) deviations in opponent color directions under different induction conditions leading to different perceptions of *hue* and *saturation*, (iii) texture masking due to the energy of the background, and (iv) texture masking due to the similarity between the features of the background and the test.

It is important to note that the properties illustrated here (properties 2, 8, 10) are examples of curves that are not standardized, as opposed to other facts in the proposed Decalogue (properties 1, 3, 4, 6, 7), in which strict comparisons (RMSE or Pearson correlation) are possible. The fact that, even in these non-standardized examples, the qualitative behavior is so compelling implies that checking the order of the curves using rank correlations is useful to quantitatively describe the alignment between artificial models and humans.

#### 3.2.1 Luminance and brightness

The first set of stimuli refers to a series of luminance-calibrated achromatic samples that illustrate the perception of brightness in backgrounds of different luminance. They illustrate the Weber law (Wyszecki and Stiles, 2000; Fairchild, 2013) and the crispening effect (Whittle, 1992), i.e., the achromatic part of Property 2 in Table 1. These effects have been related to the statistics of natural images (Laughlin, 1981; Laparra et al., 2012) and with sophisticated models of retinal adaptation (Bertalmío et al., 2020).

**Figure 5:**
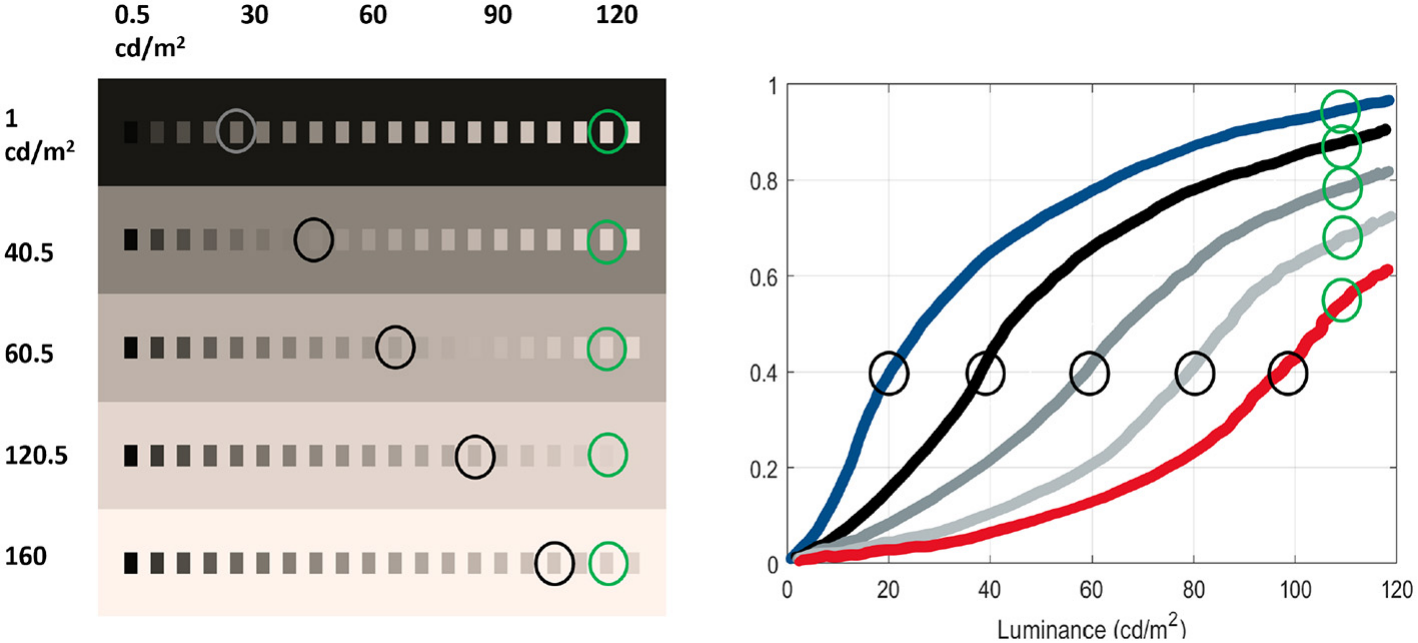
Series of stimuli eliciting non-linear brightness perception. Luminance-calibrated linearly spaced tests in different luminance-calibrated backgrounds. This illustrates the Weber law (Fairchild, 2013) as well as Whittle's crispening effect (Whittle, 1992), as summarized in Bertalmío et al. (2020).

Figure 5 shows a series of these stimuli in the (linearly spaced) range of luminance [0.5, 120] *cd*/*m*^2^ on (linearly spaced) backgrounds of luminance in the range [1, 160] *cd*/*m*^2^. These stimuli are easily generated in digital levels (i.e., ready to feed conventional artificial models) with the code provided in this work^5^, which makes use of the calibration of the Matlab toolbox Colorlab (Malo and Luque, 2002) on a standard computer screen.

Let's describe the perceived brightness of the stimuli in this test.

**First**, the series of stimuli in the darkest background clearly shows the saturation non-linearity of the brightness vs luminance curve: note that the jumps in perceived brightness for the low-luminance tests are distinctly bigger than the equivalent jumps for the same increments in luminance at the high-luminance end. In the axis of perceived brightness, the above implies that the response (blue curve) has a large slope (high sensitivity) at the low-luminance end and a saturation of such response (lower sensitivity) at the high-luminance end. That makes the *qualitative* saturating blue curve of brightness vs luminance.

**Second**, when one increases the luminance of the background (e.g., from 1 *cd*/*m*^2^ to 40 *cd*/*m*^2^), the brightness of the (same) samples is lower than in the previous series, so the *qualitative* brightness response to this second series of stimuli is below the previous one (as depicted by the *qualitative* black curve).

**Third**, by looking at the stimuli highlighted in gray in the 1 *cd*/*m*^2^ and the 40 *cd*/*m*^2^ backgrounds, it is obvious that in the brighter background, the stimuli with equivalent brightness are shifted to the right on the scale of luminance, which means that the response in black (for the stimuli in the brighter background) is shifted right-down with regard to the curve in blue (for the stimuli in the darker background). Moreover, this means that the black curve has a sigmoidal shape as it should start from zero brightness. Similar visual reasoning implies that this shift progressively increases as one increases the luminance of the background, as *qualitatively* illustrated by the samples highlighted in gray along the diagonal of the panel with the stimuli (left), leading to the shift in the curves (right).

**Fourth**, the (same) stimuli in the brightest background elicit a brightness response with a substantially different shape: the sigmoid has substantially shifted to the right (red curve), and, all in all, one can see a smooth transition of the sigmoidal response curves from the blue curve to the red curve. The crispening effect (increased sensitivity around backgrounds of similar luminance) is illustrated by the shift to the right of the points of maximum slope in the response curves.

Finally, **fifth**, the decreasing brightness of the samples of the same luminance in backgrounds of progressively greater luminance (as illustrated by the samples highlighted in green) illustrates brightness induction (Fairchild, 2013).

Of course, the *qualitative* visual observations made here do not try to substitute for the rich *quantitative* literature in which these responses are determined by accurate psychophysics (Wyszecki and Stiles, 2000; Fairchild, 2013). However, (1) the phenomena are compelling enough that one can see the qualitative trends of the curves by eye, and, as seen in the numerical experiments below, (2) these trends (visible in ready-to-use digital images) are enough to spot divergences with human behavior in certain artificial models or discriminate between models in terms of their similarity to human behavior, which is the ultimate goal of the tests presented here.

#### 3.2.2 Non-linear response to saturation and color adaptation

Responses to constant deviations from white in the red-green and yellow-blue directions of the Jameson & Hurvich color space (Hurvich and Jameson, 1957; Vila-Tomás et al., 2023) with equiluminant stimuli describe the non-linear perception of hue and saturation, as pointed out in Krauskopf and Gegenfurtner (1992) and Romero et al. (1993) in similar opponent spaces, i.e., the chromatic version of property 2 in Table 1.

**Figure 6:**
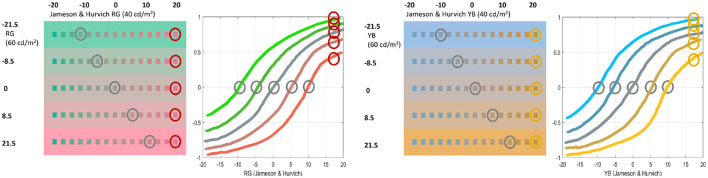
Series of non-linear perceived saturation (or response of the opponent chromatic channels) vs. linearly spaced increments in colorimetrically calibrated color opponent directions in different chromatic contexts. This illustrates the non-linear effects pointed out in Krauskopf and Gegenfurtner (1992) and Romero et al. (1993).

Figure 6 shows colorimetrically calibrated stimuli with such deviations (in the range [-20, 20] of the linear RG and YB tristimulus values of the Jameson and Hurvich space) in different backgrounds, which are easy to generate and modify by using the code provided in this work^6^. As in the previous test, let's describe the perceived hue and saturation of the stimuli to infer the qualitative shape of the responses.

**First**, take the stimuli in gray backgrounds and note that the jumps in perceived hue are bigger around the central (achromatic) stimuli than at the extremes with more saturated stimuli (either red, green, yellow, or blue): Judge the jumps in saturation close to the achromatic stimulus and at the extremes of the chromatic axes. Similarly to the responses for brightness, these differences imply a sigmoidal response to saturation when the stimuli linearly depart from white in constant steps: see the qualitative responses in gray for both the red-green and the yellow-blue directions. **Second**, these sigmoidal responses shift to the right or to the left, as can be seen from the shift of the stimuli that are perceived as achromatic in the different backgrounds (e.g., see the stimuli highlighted in gray). Note that a stimulus is seen as achromatic when the response of the mechanism tuned to red-green or yellow-blue is zero. See the corresponding shifts in the zero crossings of the sigmoids (also highlighted in gray). Finally, **third**, the shift of the responses is bigger as the saturation of the background is increased.

Again, the goal of this test is not to substitute the original accurate psychophysics done on humans (Krauskopf and Gegenfurtner, 1992; Romero et al., 1993) to point out these phenomena. On the contrary, they just represent an easy way to get digital images that can be used to test artificial models and check if their responses qualitatively behave like humans.

#### 3.2.3 Texture masking 1 (energy): non-linear adaptive contrast response

The same kind of qualitative derivation of human-like responses can be applied to the perceived contrast of textured patterns with calibrated frequency content and controlled luminance. The test presented here illustrates the fact that perceived contrast non-linearly depends on linearly increasing Michelson contrast (Legge and Foley, 1980; Legge, 1981) and this response decreases with (is masked by) the energy of a background of similar texture (Foley, 1994; Watson and Solomon, 1997). This corresponds to property 8 in Table 1. The stimuli presented in the following example can be reproduced and modified both in frequency orientation, average luminance, and contrast with the code provided^7^.

**Figure 7:**
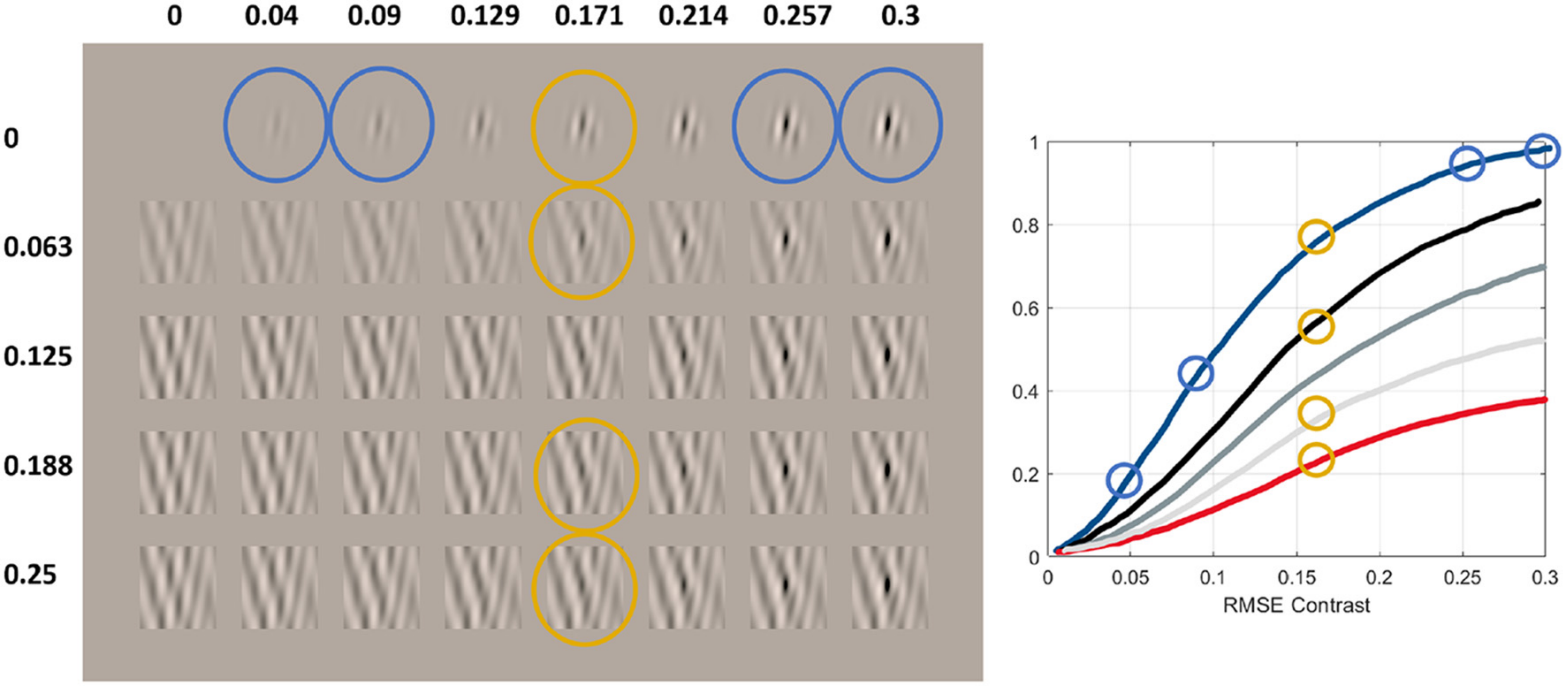
Series of non-linearly perceived contrast (or response of the mechanisms tuned to certain texture) vs. linearly spaced increments in contrast-calibrated test of controlled spatial frequency in backgrounds of different (controlled) energy. This illustrates the non-linear effects pointed out in Georgeson and Sullivan (1975), Legge and Foley (1980), Legge (1981), and Daly (1990).

Figure 7 shows Gaussian-windowed test noise patches of 4 cycles/degree (cpd) in images subtending 1 degree with an average luminance of 50 *cd*/*m*^2^ and linearly spaced RMSE contrasts (from left to right) in the range [0, 0.3]. The different rows show the same tests on different backgrounds of noise of 4 cpd with linearly spaced RMSE contrast in the range [0, 0.25].

Similarly to the previous cases, let us describe the perceived contrast along the two dimensions of the panel: test: left to right, and background: top to bottom. Again, the qualitative shape of the responses will be determined by the perceived jumps of contrast of the tests (from left to right) and by their variation as one increases the energy of the background (from top to bottom).

**First**, for the zero-contrast background (first, top row), the jumps in perceived contrast in the low-contrast end (left) are bigger than the jumps in perceived contrast in the high-contrast end (right). See the differences in perceived contrast in the tests highlighted in blue. This implies a saturating contrast response curve (as in the previous examples), i.e., the blue curve.

**Second**, as the contrast of the background is increased (see stimuli highlighted in orange), the perceived contrast of the test is reduced. This implies that subsequent curves (black and lighter shades of gray) are below the initial blue curve.

Finally, **third**, in order to perceive the tests with equivalent contrasts in backgrounds of progressively greater energy, the necessary contrast of the test increases; this means that the sigmoidal curves shift to the right.

As in the previous examples, the qualitative behavior illustrated by this series of digital images generated by our code should give (in artificial models) corresponding saturating curves with smooth variation from the blue (zero-contrast background) condition to the red (high-contrast background) condition, and hence the lower response curve.

#### 3.2.4 Texture masking 2 (features): interaction between orientations

**Figure 8:**
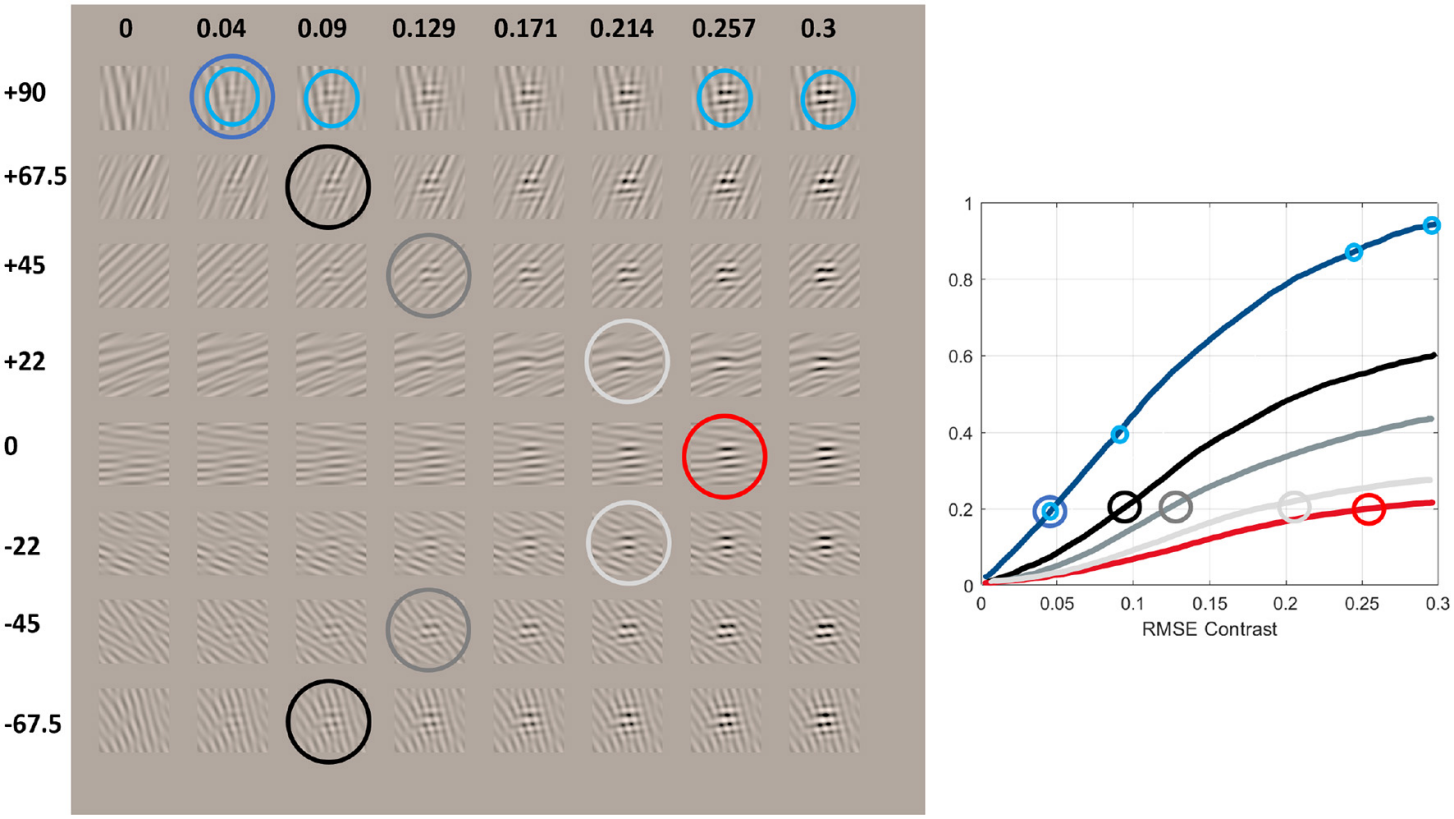
Series of non-linearly perceived contrast (or response of the mechanisms tuned to certain texture) vs. linearly spaced increments in contrast-calibrated tests of controlled spatial frequency in backgrounds of different orientations. This illustrates the non-linear effects pointed out in Foley (1994) and Watson and Solomon (1997).

Reduction of sensitivity (the so-called masking) also happens when a certain test is presented on top of a background that shares some feature with the test (Ross et al., 1991; Foley, 1994; Watson and Solomon, 1997), i.e., properties 9 and 10 in Table 1. The next example, Figure 8, refers to the specific case of interaction between orientations of test and background. It can be reproduced and modified in terms of frequency, orientation, contrast, and average luminance with the code provided^8^.

Figure 8 shows 6 cpd horizontal Gabor patches with an average luminance of 50 *cd*/*m*^2^ and RMSE contrast increasing linearly from left to right in the range [0, 0.3]. These Gabor patches are shown on top of band-pass noise of contrast 0.2, with the same frequency, but different orientation. The numbers in the different rows show the angular difference between tests and backgrounds. The figure shows some compelling facts that lead to clear qualitative trends in the response curves.

**First**, the test is better seen (has bigger visibility or perceived contrast) when the background is orthogonal to the test (in the first row). In that row, the different jumps in visibility in the low-contrast and high-contrast ends (tests highlighted in cyan) indicate a saturating response, as in the previous examples (response curve in blue).

**Second**, the necessary contrast to detect the test smoothly increases as the difference in orientation between test and background decreases: see that the tests highlighted in blue, black, shades of gray, and red approximately have the same visibility over the different backgrounds with angular differences in the range [90,0] deg. The trend is similar for negative angular differences. This implies a smooth variation (decrease) of the response curves in terms of the difference between test and background.

**Third**, the biggest masking is obtained when test and background are aligned (the red curve is clearly the lowest response curve). This and the previous fact imply that the general trend is this smooth transition of the non-linear curves from the blue to the red.

### 3.3 Proposed methodology

Our proposal is simple: use the code provided here^9^ to generate the stimuli (digital images well-calibrated in luminance, color, and spatio-temporal frequency) that illustrate the 10 compelling properties listed in Table 1 describing the adaptive information bottleneck of low-level human vision. The resulting digital images are organized in series that correspond to progressive stimulation of a vision system in particular ways. The interesting point is that this set of controlled stimulation conditions leads to intuitive responses (as shown above), or even to standardized sensitivity curves or surfaces that are also provided with the code see Section 1 of Supplementary material.

Once stimuli are generated, they are used to feed any artificial image-computable model. Then, depending on the model, the user decides where to read from the network under consideration and the read-out mechanism to get *visibility* values to generate artificial series of response curves.

The resulting curves can be qualitatively assessed by checking the correspondence of their shape with our own visual experience as in the examples above. However, in order to simplify the (blind-quantitative) use by the non-expert, we propose two different correlation measures for the cases where experimental ground truth is (or is not) available:

- **Pearson Correlation:** in the case of properties where clear ground truth is available, we propose to quantify the alignment using Pearson correlation between the ground truth and the predictions of the models. Pearson correlation is insensitive to a global (arbitrary) scale of the response, and this is generally acceptable because a single global scale is not important. In order to reduce the relevance of this arbitrary scale, we propose to evaluate a single Pearson correlation measure for groups of comparable curves (for instance, response curves for different frequencies but the same contrast stimulation–e.g., in Props. 6–8-, or curves with known scaling between the achromatic and chromatic response–e.g., in spectral sensitivities in Prop. 1, CSFs in Props. 3 and 4, or scale of achromatic and chromatic curves in Props. 6 and 7). In those cases, by evaluating the Pearson correlation for groups of curves, it captures if they are scaled with the proper relative size (which should be reproduced by the models). Using the ground truth curves shown in Supplementary material (and associated code), the Pearson correlation can be computed for about 70% of the proposed tests (Props. 1, 3–7, the cases with no adaptation in Prop. 2, and also the no-masking curves in Props. 8–10).
- **Rank correlation:** In the cases of brightness and chromatic adaptation (Prop. 2) and cases where there is spatial masking with the same stimulus (Prop. 8) or with different stimuli (Props. 9 and 10), direct visualization of the tests shows the trend for the variation of the curves (mainly shifting and attenuation of the curves when increasing the strength of the adaptor–either luminance or saturation of the background or illuminant or contrast of the masker). The specialized literature describes these trends, but in too sparse (or not comparable) situations that are not properly captured by the (ready for ANNs) stimuli in the database. In this situation, Pearson correlation is not applicable. However, the relative order (or rank) between the curves for different strengths of the adaptor is known. Reproduction of this known rank of curves at several locations of the abscissas is possible using rank correlation (e.g., Spearman or Kendall correlations)to obtain a quantitative descriptor per Property. Although necessary in cases where no experimental data is available, this kind of descriptor of “ranking of curves” can also be used in cases with ground truth because Pearson correlation may miss (or not clearly capture) the rank between the curves, which usually is a qualitative trend that should be reproduced by the models.

Choosing an appropriate layer to read from and a read-out mechanism can be a crucial step because, as it happens in the human-visual system, certain behaviors are expected to be happening at different points of the visual processing pipeline. BrainScore (Schrimpf et al., 2018) proposes using an independent test set to evaluate how each layer of an artificial model matches to certain areas of the brain. Then, it's only a matter of reading from the layer that has a better match with the behavior we want to measure. As a different example, within this work we employ models that have been designed to accommodate certain parts of the human visual system at certain layers, so choosing the appropriate layer to read from is straightforward. We also provide a set of control experiments (Supplementary material; Section 2) that showcase how does the read-out mechanism affect the obtained results so that the interested reader can make an informed choice.

In the case of properties 2, 8, 9 and 10 these curves have to be compared with the kind of qualitative curves described above, which given the clarity of the selected stimuli can be drawn by simple visual observation of the stimuli as described above.

In the case of property 5 [existence of center-surround and Gabor-like receptive fields tuned to achromatic, red-green and yellow-blue patterns (Shapley and Hawken, 2011)], the more straightforward method is checking their presence by reading the response to deltas from single neurons or from the Jacobian of the network at that layer (Martinez et al., 2018; Gomez-Villa et al., 2020b; Li et al., 2022). Other indirect methods could be (1) using reverse correlation feeding the network with controlled noise [also generable using Vistalab (Malo and Gutierrez, 2002) following the appropriate literature (Eckstein and Ahumada, 2002)], or (2) using artificial psychophysics based on adaptation [e.g., the Blakemore and Campbell experiment (Blakemore and Campbell, 1969)]. However, this very last method to measure proeprty 5 relies on fulfillment of adaptation proeprties 6–10, which may not hold in non-human networks.

In the above (non-standardized) cases the general trends of the curves can be qualitatively assessed in detail: general shape of the curves, the blue response and the red curve being the biggest and the lowest, respectively, and the transition from one to the other. Note that user of the provided code can change the parameters of the stimuli and infer new curves by applying a similar visual analysis. For the receptive fields they can be analyzed using shape parameters in the spatial or the Fourier domain as classically done in visual neuroscience (Ringach, 2002; Ringach and Shapley, 2004; Martinez et al., 2017; Loxley, 2017) and the same for the chromatic tuning in standard color spaces (Tailby et al., 2008; Gutmann et al., 2014; Gomez-Villa et al., 2020b).

Finally in the case of sensitivity curves or surfaces which are standardized or available in the code (proeprties 1, 3, 4, 6 and 7) the visibility values obtained from the models can be numerically compared with the provided ground truth.

The above quantitative descriptors clearly display some limitations. First, they obviously overlook relevant qualitative properties of the results. For example: (a) Correlations do not capture the change of slope happening in crispening and chromatic adaptation in Prop. 2. This change of slopes is relevant to decide between models (Laparra et al., 2012; Bertalmío et al., 2020). More complicated descriptors of the qualitative shape of these specific curves could be developed, but they are out of the scope of this work. (b) Assessment of receptive fields in certain layers is done by the eye in many influential works (Olshausen and Field, 1996; Bell and Sejnowski, 1997; Hyvarinen et al., 2009; Krizhevsky et al., 2012a) that report the emergence of Gabor-like receptive fields and their similarity with biological receptive fields in V1 just by inspection of the filters. Analysis of the spatio-frequency properties and chromatic properties of the receptive fields, as in Gutmann et al. (2014); Martinez et al. (2017); Loxley (2017); Gomez-Villa et al. (2020b) comparing with (Ringach, 2002) and Tailby et al. (2008), is also possible but more unusual.

Second, the derivation of a single score per model as the average of scores over experiments [as the compromise solution taken in BrainScore (Schrimpf et al., 2018)] is arguable. The selection of properties to average over is always somewhat arbitrary, and alternative selections lead to global scores with different variances (and discriminative power). Supplementary material illustrates this limitation by showing bootstrap examples in the set of quantitative results shown below.

**Figure 9:**
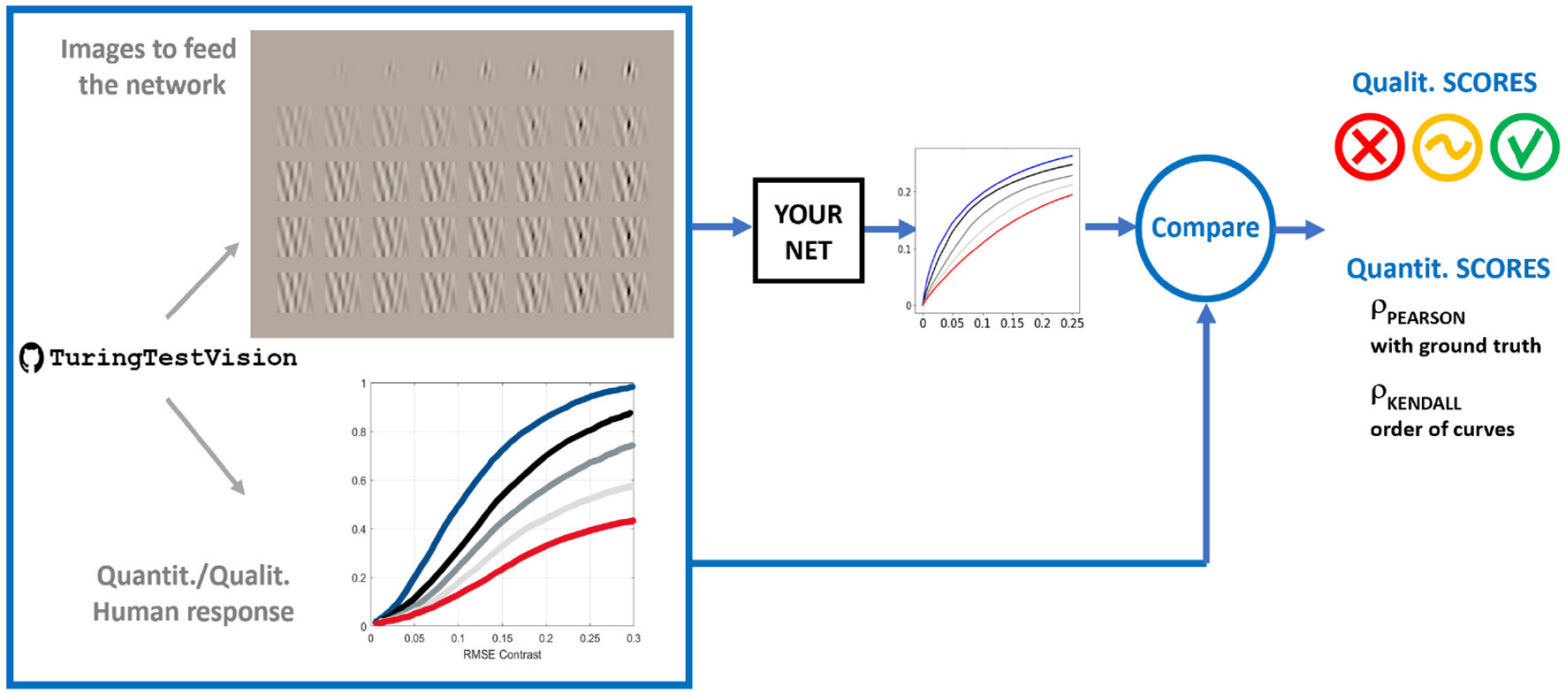
The proposed method: feed the model with a series of images, compute responses (using the simplest possible read-out mechanism), and make quantitative comparisons with standard sensitivity surfaces or qualitative comparisons checking the non-linearity using different adaptation conditions.

This qualitative/quantitative methodology is summarized in Figure 9 and applied in the next experimental section for three illustrative networks.

## 4 Experiments: analysis of three illustrative deep models

### 4.1 Networks and experimental setting

In our experiments, we check the behavior on the proposed *Decalogue* of three recent networks of similar architecture:

1. A parametric vision model, the ***BioMultiLayer*** network (Martinez et al., 2018), which consists of a cascade of four linear+non-linear stages that account for (1) color opponency and adaptation, (2) contrast computation, (3) contrast sensitivities and energy masking, and (4) wavelet analysis and cross-masking between textures. The linear parts of all the stages were not optimized, but they were directly inspired by classical psychophysical or physiological literature. The non-linear parts were implemented via divisive normalization (Carandini and Heeger, 1994; Malo et al., 2006; Laparra et al., 2010; Malo et al., 2024; Carandini and Heeger, 2012). The non-linearities of the 2nd and 3rd stages of the model were tuned via the psychophysical method of maximum differentiation in Malo and Simoncelli (2015). The non-linear parts of the 1st and 4th stages were tuned to reproduce subjective opinions on distortion and contrast masking facts (Martinez et al., 2018, 2019). The statistical properties of the model and its relations with recurrent models were studied in Gomez-Villa et al. (2020a) and Malo et al. (2024), respectively.
2. A non-parametric model to predict subjective image quality, the ***PerceptNet*** (Hepburn et al., 2020), which starts with a non-linear front-end at the retina followed by a cascade of three linear+non-linear stages. The architecture was intended to accommodate similar vision facts that motivated the *BioMultiLayer*. The *PerceptNet* architecture is similar to AlexNet (Krizhevsky et al., 2012b) but its non-linearities were formulated using an end-to-end optimizable divisive normalization (Laparra et al., 2017; Ballé et al., 2017). Both the linear and the non-linear parts of *PerceptNet* were end-to-end tuned to maximize the correlation with humans on subjective image distortions (Hepburn et al., 2020). Non-parametric layers of *PerceptNet* are not easy to interpret, as pointed out recently (Vila-Tomás et al., 2025).
3. An image segmentation model, the ***Bio U-Net*** (Hernández-Cámara et al., 2023), with the same style encoder as the non-parametric *PerceptNet* (a cascade of linear + divisive normalization stages), but augmented with a decoder that recovers the original dimension of the input signal and predicts a class per pixel for semantic segmentation. The encoder and the decoder were tuned to optimize segmentation in different databases. The benefits of the biologically inspired non-linearities of this model for segmentation have been further studied thereafter (Hernández-Cámara et al., 2025b).

We assumed a visual field of 2 degrees with a sampling frequency of 64 cycles/deg, i.e., we fed the models with 128 × 128 images. We measured the responses of the models to specific tests through the Euclidean departure between the response to test+background with regard to the response to the isolated background.

### 4.2 Results I: qualitative analysis

In this section, we qualitatively assess the results by checking the shape and relative scales of the curves obtained by the models in relation to the observations made in Section 3.2. In the next section, we apply the quantification of the alignment proposed in Section 3.3.

#### 4.2.1 Spectral sensitivities and color responses (properties 1 and 2)

**Figure 10:**
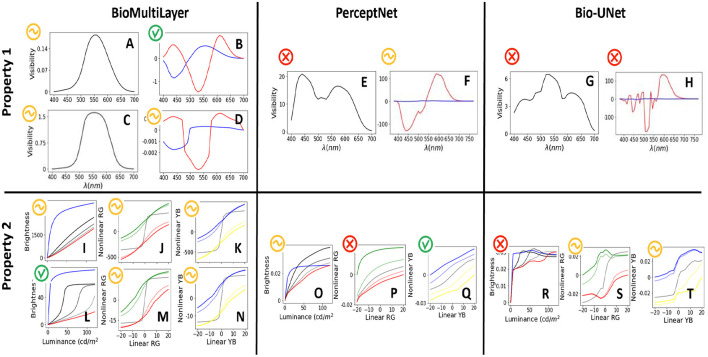
Spectral sensitivities of the considered models **(top)** and corresponding responses to luminance and linear deviations from white in the cardinal red-green and yellow-blue directions **(bottom)**. Subplots **(A)**, **(C)**, **(E)**, and **(G)** display the achromatic spectral sensitivity for the models, while subplots **(B)**, **(D)**, **(F)**, and **(H)** display the chromatic sensitivity obtained through hue cancellation (Vila-Tomás et al., 2023). Knowledge of the standard spectral sensitivity, the CIE *V*_λ_ curve (Wyszecki and Stiles, 2000), or the standard spectral sensitivity of the opponent channels (Hurvich and Jameson, 1957) indicates which model is correct in Prop. 1. Subplots **(I)**, **(L)**, **(O)**, and **(R)** show the brightness responses to luminance. Subplots **(J)**, **(K)**, **(M)**, **(N)**, **(P)**, **(Q)**, **(S)**, and **(T)** show the responses to deviations from white. In this case, the stimuli proposed here (Figures 5, 6) and the associated human behavior described above indicate the correct trends in the responses of Prop. 2.

Figure 10-top shows the response of the models to quasi-monochromatic stimuli^10^ to get the spectral sensitivity of the neurons (property 1). In order to point out the relevance of the layer from which responses are measured, in the case of the *BioMultiLayer* network, we consider direct read-out of the response (with sign) in the first linear layer (subplots A and B) and in the last non-linear layer (subplots C and D). In this network, the first linear layer has achromatic and opponent channels defined by construction, so the *V*_λ_ (Wyszecki and Stiles, 2000) (subplot A) and the opponent curves of Jameson & Hurvich (Hurvich and Jameson, 1957) (subplot B) are trivially obtained. Interestingly, the spectral sensitivities at the last non-linear layer are wide-band positive in the first channel of the network and opponent in the other two channels, but their shapes are substantially modified with regard to the human-like behavior at the first layer. These differences and the uneven relative scaling between the achromatic and chromatic responses justify the qualitative scores given in each case. We can conclude that spectral sensitivity in this model is human-like at the front end but degrades throughout the network. In other words, as suggested in Section 2.2, read-out location matters, and a certain kind of information should be extracted from a specific place in the model.

The *PerceptNet* has a color space change after the retinal non-linearity. We measure the spectral sensitivity at that point because the design assumed that achromatic and chromatic channels could emerge there. Results show that the first channel displays an all-positive but bimodal response (subplot E), and for the other two channels, one of them certainly displays opponent-like responses, but the other is basically insensitive (subplot F).

The very same location of the encoder of the segmentation *Bio-U-Net* has very different sensitivities despite it having the same architecture as the *PerceptNet* up to that layer. The sensitivity of the (supposedly) achromatic channel is very noisy, and the other two channels are clearly non-human (subplots G and H).

On the other hand, Figure 10-bottom checks property 2 by showing the responses to (i) luminance and to deviations from white in the (ii) red-green and (iii) yellow-blue directions (left, center, and right, respectively). In the achromatic case, tests in the range [0.5, 120] *cd*/*m*^2^ are shown on top of backgrounds of different luminance in the range [1, 160] *cd*/*m*^2^. The response curves in different backgrounds are depicted in blue, black, and progressively lighter shades of gray until red, as in Figure 5. In the chromatic cases, responses are computed with tests on an achromatic background (black curve) and on backgrounds of progressively saturated color (reddish and greenish curves, and bluish and yellowish curves as in Figure 6).

For the *BioMultiLayer* model, we have such responses for two different layers: first (I, J, K) and fourth (L, M, and N). The achromatic response of the first layer is certainly non-linear for the darkest background, and the response gets attenuated when the luminance of the background is increased (see the transition from curves in blue to red in subplot I). However, these responses do not reproduce the crispening (sigmoids shifting to high luminance), and responses for high luminance backgrounds are too linear. As a result, the achromatic behavior of this layer has been qualified as non-human. The chromatic responses display sigmoidal shapes, and they shift in the right directions under different backgrounds (subplots J and K). However, the non-linearities for the chromatic backgrounds are very smooth compared to the sharpness of the non-linearity for the achromatic background. As a result, the human similarity of chromatic behaviors has been qualified as intermediate. In contrast, the achromatic response of the fourth layer (subplot L) does reproduce the non-linear behavior and crispening, so it has been qualified as more human-like than the achromatic response of the first layer. Shifts of the chromatic non-linearities are stronger depending on the background, but the non-linearities in achromatic backgrounds (black curves in subplots M and N) are still too sharp. Therefore, the score remains the same.

The *PerceptNet* model displays non-linear behavior and crispening in the responses to the achromatic series (subplot O). However, note how the curves corresponding to light backgrounds exceed the response on dark backgrounds, so human similarity has been qualified as intermediate. The responses to red-green series in *PerceptNet* shift in the right directions on different backgrounds, but they are too linear (and hence wrong) in subplot P. In contrast, the blue-yellow responses (subplot Q) display a rather human behavior.

Finally, the *Bio-U-Net* shows a clearly non-human achromatic response: note the noise and wrong order in the curves with no trace of crispening (subplot R). In contrast, the responses to the chromatic series display the expected sigmoidal shape with the shift in the proper directions for the different chromatic backgrounds (subplots S and T). Noisy and unstable responses are what determined the intermediate score.

#### 4.2.2 Achromatic and chromatic contrast sensitivities and receptive fields (props. 3, 4, and 5)

**Figure 11:**
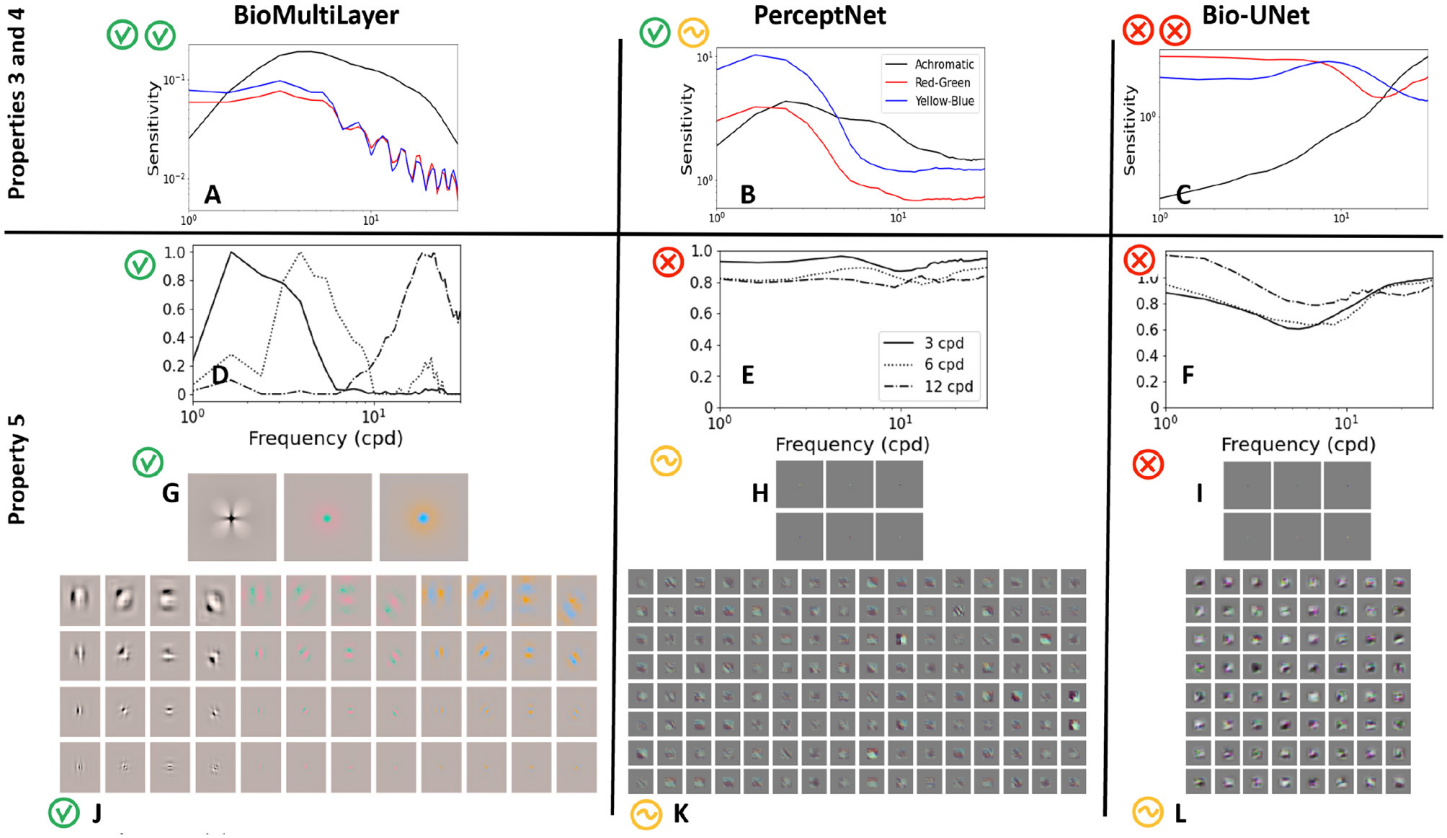
Achromatic and chromatic CSFs of the considered models [top subplots **(A–C)**]. Subplots **(D–F)** show the receptive fields tuned to 3, 6, and 12 cpd (different line styles) computed for the different models using the Blakemore & Campbell CSF adaptation method (Blakemore and Campbell, 1969). Subplots **(G–I)** show the receptive fields obtained using delta stimuli (Martinez et al., 2018) at early layers of the models and subplots **(J–L)** at late layers.

The top row of Figure 11 shows the achromatic Contrast Sensitivity Function (property 3, black curve) and the red-green and yellow-blue Contrast Sensitivity Functions (property 4, red and blue curves, respectively). These CSFs have been computed from the responses to noise patterns of controlled spatial frequency and the same low contrast (*C*_RMSE_ = 0.05) for every frequency. Patterns were generated in the corresponding color channel of the Jameson & Hurvich color space (Hurvich and Jameson, 1957) that isolates luminance, red–green, and yellow–blue components. We consider the responses at the last layer of the networks, and we plot the Euclidean distance between the responses for each pattern and for a flat image of the same average color.

The CSFs of the *BioMultiLayer* model (subplot A) strongly resemble the human CSFs (Campbell and Robson, 1968; Mullen, 1985): the achromatic response is band-pass with peak sensitivity around 4 cpd and high cut-off frequency (above 32 cpd), and the chromatic responses are lower and basically low-pass with cut-off frequencies of about 15 cpd.

The achromatic CSF of the *PerceptNet* is also band-pass (black curve in subplot B), but the chromatic CSFs are far from human because their shape is also band-pass and the responses to modulations in the YB direction are much bigger than the responses to equivalent achromatic modulations.

Finally, the *Bio-U-Net* (subplot C) displays a strongly non-human behavior: see the non-plausible high-pass behavior of the responses to achromatic gratings, and the bigger responses to chromatic gratings in the mid-frequency range.

Supplementary material makes a systematic study of the impact on the CSFs of different read-out locations along the three models and six different read-out strategies, thus illustrating the points made in Section 2.2.

The receptive fields of the models (property 5) have been proved in two ways: (1) a *psychophysical* method based on the Blakemore & Campbell experiment (Blakemore and Campbell, 1969), which relies on the attenuation of the CSF under adaptation for different frequencies, and (2) a *physiological* method based on recording the response to deltas in the luminance, red–green, and yellow–blue channels (Martinez et al., 2018). This is another example of two different experimental settings (psychophysical and physiological) to measure the alignment mentioned in Section 2.2.

In the *BioMultiLayer* model, the attenuation of the achromatic CSF when the gratings are shown on top of backgrounds of specific frequencies (subplot D) reveals the existence of narrow-band sensors with bandwidth that increases with frequency, which is consistent with human behavior (Blakemore and Campbell, 1969; Simoncelli and Adelson, 1990). This comes from the fact that the linear part of the 4th layer of this model is made of wavelet kernels, and their response is non-linearly attenuated by the activity of neighbor sensors tuned to the same feature through divisive normalization.

On the other hand, when checking the shape of the receptive fields using delta functions, one gets two biologically plausible results: (a) in the 3rd layer of the *BioMultiLayer* network, receptive fields are center-surround patterns in the achromatic, red–green, and yellow–blue directions (subplot G), and (b) in the fourth layer, one gets local frequency filters with different orientations and scales (subplot J) as happens in biological vision at LGN (Cai et al., 1997; Shapley and Hawken, 2011) and V1 (Hubel et al., 1959; Watson, 1983).

For *PerceptNet*, results are quite different: first, the Blakemore and Campbell experiment shows non-human wide-band mechanisms (subplot E). This is not only due to the non-human nature of the CSFs, it also means that any frequency leads to attenuation of the responses to patterns of any frequency. This departure from human behavior is also visible when getting the receptive fields from the last layer of the network using deltas: one gets oriented filters, but all with the same size and with low-frequency blobs. Moreover, chromatic information is spread along all the filters (subplot K), as opposed to what happens in the early layers, where one gets achromatic, red–green, and yellow–blue responses (subplot H).

Finally, in the *Bio-U-Net* model, the Blakemore and Campbell experiment also leads to non-human ratios of the CSFs (subplot F). The receptive fields obtained from deltas in the first layers lead to center-surround blobs but not in definite chromatic directions (subplot I). In the central layers of the encoder, one gets larger receptive fields which have no clear spatial oscillations nor preferred chromatic directions (subplot L).

#### 4.2.3 Contrast saturation, dependence on frequency (properties 6 and 7)

**Figure 12:**
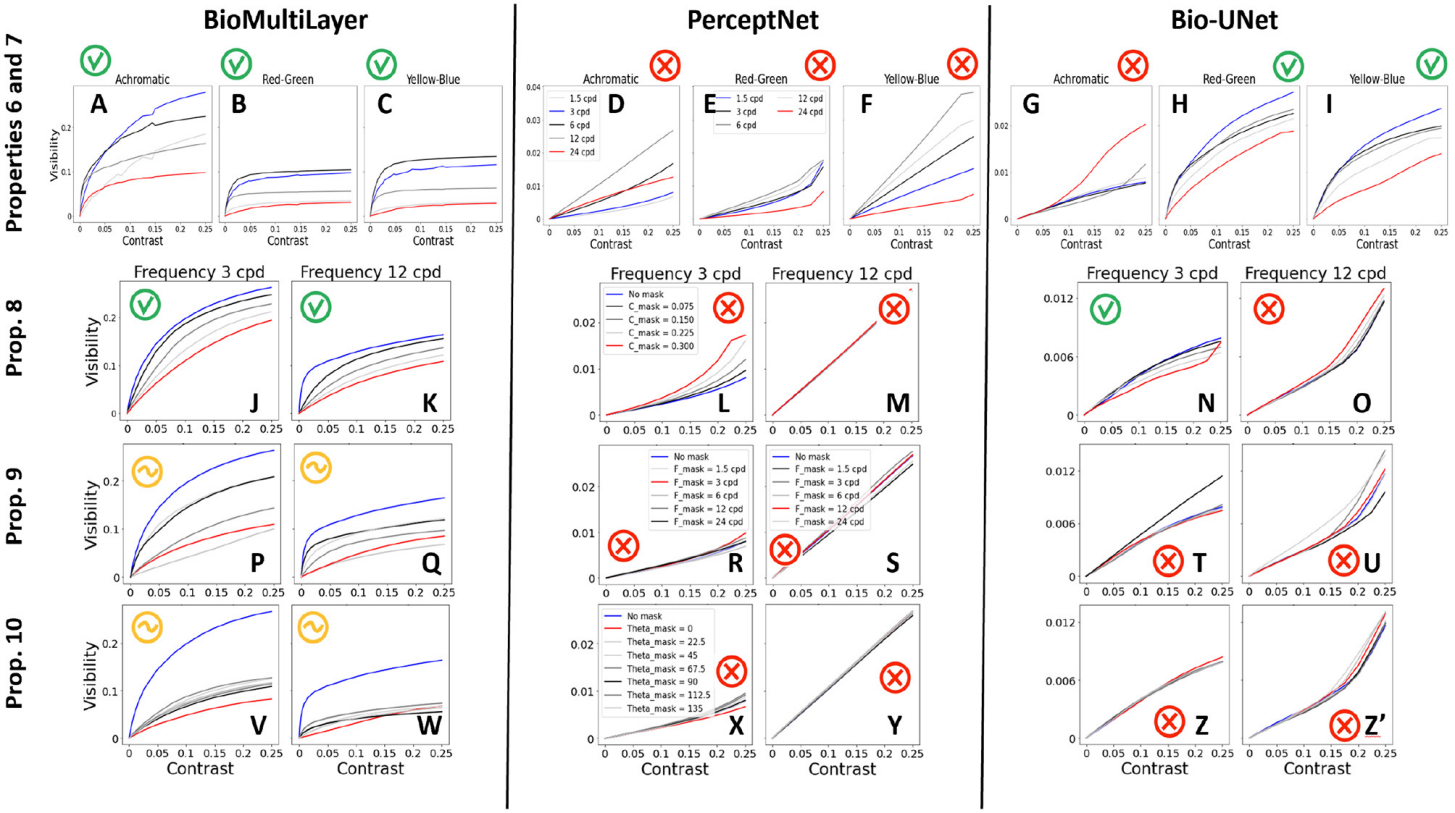
Contrast responses of the considered models in different masking conditions and for achromatic and chromatic textures of different frequencies. The color code (indicated in the subplots corresponding to Perceptnet, but applicable to the equivalent curves of the other models) has been designed so that human response curves would be in the blue-red order, as in Figures 7, 8. In this way, it is obvious which model reproduces human behavior better.

The top row of Figure 12 shows the visibility response (Euclidean difference of response with respect to the response of a uniform gray image) for achromatic patterns of different frequencies and for red-green and yellow-blue patterns of different frequencies, all seen in isolation (properties 6 and 7). Line styles for the different frequencies in the achromatic and chromatic cases are different according to the different order expected for band-pass and low-pass systems. In every case, a match with human behavior would be illustrated by having the blue curve at the top and the red curve at the bottom, with a smooth transition from black to light gray in between.

The *BioMultiLayer* model leads to saturating responses with larger intensities in the achromatic case (left) than in the chromatic cases (subplots A and B-C), as in humans (Watson and Solomon, 1997; Martinez-Uriegas, 1997). The achromatic response to mid-frequency (3 cpd, in blue) is clearly bigger than the response to the other frequencies, which is smoothly reduced for higher frequencies (from 6 to 24 cpd) and also attenuated for 1.5cpd. On the other hand, the chromatic responses are basically ordered according to frequency in a low-pass fashion. All these trends are in good agreement with human behavior.

The achromatic responses of the *PerceptNet*, though band-pass, exhibit quite a linear, non-saturating or even expanding behavior (subplot D). Moreover, these achromatic responses are not bigger than the response to chromatic patterns, particularly the yellow-blue (subplot F), which is contrary to human perception.

The responses for the chromatic patterns in the *Bio-U-Net* model exhibit human-like saturation, and they are in the right frequency order (subplots H and I), but they are larger than the responses for achromatic patterns (subplot G), which is contrary to human perception.

#### 4.2.4 Energy masking and feature masking (properties 8–10)

Each panel of the second row in Figure 12 shows the responses to a 3 cpd achromatic pattern (left) and a 12 cpd achromatic pattern (right) seen on top of a mask (noise of the same frequency and orientation) with progressively larger RMSE contrast (in the range [0,0.3]) leading to different response curves in different colors (from blue to red), thus checking the effect of the energy of the background (prop. 8). The color code has been selected so that the no-mask case is depicted in blue (less attenuated in humans), and colors from black to light gray and red are taken for progressively bigger contrasts of the mask.

The responses of the *BioMultiLayer* in Figure 12 (subplots J, K) progressively attenuate as the energy of the background is increased, in line with the reduction in visibility of the test shown in each column of Figure 7. And this happens both for low and high frequency, with bigger responses for the mid-frequency. Therefore, the behavior is qualitatively human. The *PerceptNet* displays a completely non-human behavior: for the 3 cpd tests, progressively larger masks induce enhancement of the expansive (non-saturating) response, and the responses for the high-frequency patterns are larger, linear, and do not show significant variation with the mask. Finally, the *Bio-U-Net* model does display human-like attenuation of the response to 3 cpd patterns (subplot N). However, the responses to 12 cpd patterns (subplot O) are not human-like because of their (large) size, expansive shape, and increase with the energy of the mask.

The panels of the third row of Figure 12 show the responses for an achromatic test of 3 cpd (left) and 12 cpd (right) seen on top of backgrounds of different frequencies (and 0.2 contrast) compared to the no-mask condition, i.e., it checks the frequency cross-masking (property 9). The color code has been selected so that the no-mask case is depicted in blue (less attenuated in humans), and colors from black to light gray and red are taken for progressively closer frequencies in mask and test, which lead to increased attenuation of response in humans.

The response of the *BioMultiLayer* model is bigger in the no-mask condition, displays substantial attenuation when the background shares the same frequency as the test (red curves in subplots P and Q), and responses are bigger for 3 cpd than for 12 cpd. In each case, the optimal frequency is not the one that leads to the biggest attenuation, but it is close to it. The *PerceptNet* responses are not human because for the low frequency, subplot R, responses are not saturating regardless of the mask, and the responses for high frequency are larger, linear, and the presence of backgrounds leads to larger responses (subplot S). The *Bio-U-Net* does not show human-like trends because in the case that displays a saturating response, the presence of a background leads to responses larger than in the no-mask case (black curve in subplot T). The behavior in subplot U is non-human for the same reasons stated in subplots N and O.

Finally, the last row of Figure 12 shows the responses for low- and high-frequency achromatic patterns (left and right, respectively) seen on top of backgrounds of the same frequency but different orientations; i.e., it checks the orientation cross-masking (property 10). Again, the color code has been chosen so that in a human, the blue curve would be at the top and the red would be at the bottom, as in Figure 8.

For this last example, the *BioMultiLayer* model gets bigger attenuation for the background of the same orientation, particularly for high frequency (see the red curves), and the other orientations lead to responses that are between the no-mask condition (in blue) and the same-orientation background (in red) in subplots V and W. The other models give clearly non-human results because (on top of the arguments used in previous cases) stimulation on backgrounds of the same orientation (red curves) does not lead to the expected attenuation, and bigger attenuation is obtained for backgrounds that are almost orthogonal to the test, which is not what humans experience in Figure 8.

#### 4.2.5 Summary of qualitative results

The qualitative evaluation of the considered models over the proposed tests is summarized in Table 2. From this table, there is a clear ranking of the alignment between the models and humans. It is not surprising that the parametric model (the *BioMultiLayer*) has greater alignment in the linear parts (props. 1 and 5) since sensitivities and center-surround and Gabor receptive fields were parametrically built into that model.

**Table 2:** Summary of qualitative results, which for these models, is enough to clearly identify the best alignment.

	**Facts / properties**	**BioMultiLayer**	**PerceptNet**	**Bio-UNet**
1	Spectral sensitivities (achromatic and opponent)	**~**✓	**×~**	××
2	Brightness & Color response saturation	✓**~**	**~** **~**	**×~**
3	Achromatic contrast sensitivity (bandwidth)	✓	✓	×
4	Chromatic Contrast Sensitivity (Bandwidth)	✓	**~**	×
5	Spatio-chromatic receptive fields	✓✓✓	**×~** **~**	**××~**
6	Non-linear contrast response: saturation	✓ ✓	××	×✓
7	Non-linear contrast response: frequency order	✓	×	**~**
8	Context effects: energy	✓	×	**~**
9	Context effects: frequency	**~**	×	×
10	Context effects: orientation	**~**	×	×

More interestingly, the band-pass behavior of the sensors emerged from modifications in the CSFs in our simulation of the Blakemore and Campbell experiment. It is also interesting that the close reproduction of the band-pass and low-pass behavior and the relative scaling of the CSFs obtained from responses to sinusoids (an original check done here) was not built in. This indicates that the (non-trivial) gain of the center-surround cells and the Gabor cells was properly adjusted through the indirect psychophysical experiments done to set their parameters. As a result, the relative order of the (saturated) frequency responses (prop. 7) is also ok, both for achromatic and chromatic textures. The saturation of the responses to Gabor stimuli in isolation (prop. 6) is better reproduced in the parametric model than in the Bio-UNet. The difference between them is more evident when one digs deeper using props. 8-10 because they need proper interaction between texture sensors, and this was only easy to do in a parametric model such as the *BioMultiLayer*.

However, note that the reproduction of the interaction between features (both in color, property 2, and in texture, properties 9 and 10) is not properly reproduced, not even in the *BioMultiLayer*, pointing out that more work is needed to adjust its parameters, as discussed below.

According to the proposed test, the other two models (the non-parametric *PerceptNet* and the *Bio-U-Net*) are *less human*, in that order of alignment. This also makes sense because the *PerceptNet* was tuned to reproduce low-level human opinion on distortion, while the *Bio-U-Net* was just tuned to reproduce a specific mid-level vision goal such as image segmentation. In the discussion, we elaborate more on the combination of goals that may explain the organization of the visual system.

In any case, we see that even with this qualitative application of the proposed test (again, quantitative comparisons could be done with properties 1, 3, 4, and 6, even for moving patterns), a significant ranking is possible, and, as discussed below, the qualitative behaviors, when they are properly understood, suggest significant changes in the architectures and training of the models. Quantitative automation of the optimization should be iteratively done by alternating goals of different natures, as suggested in (Martinez et al. 2019): optimize for conventional goals and then fine-tune to reproduce the effects pointed out by the test proposed here (or the other way around).

### 4.3 Results II: quantitative analysis

**Figure 13:**
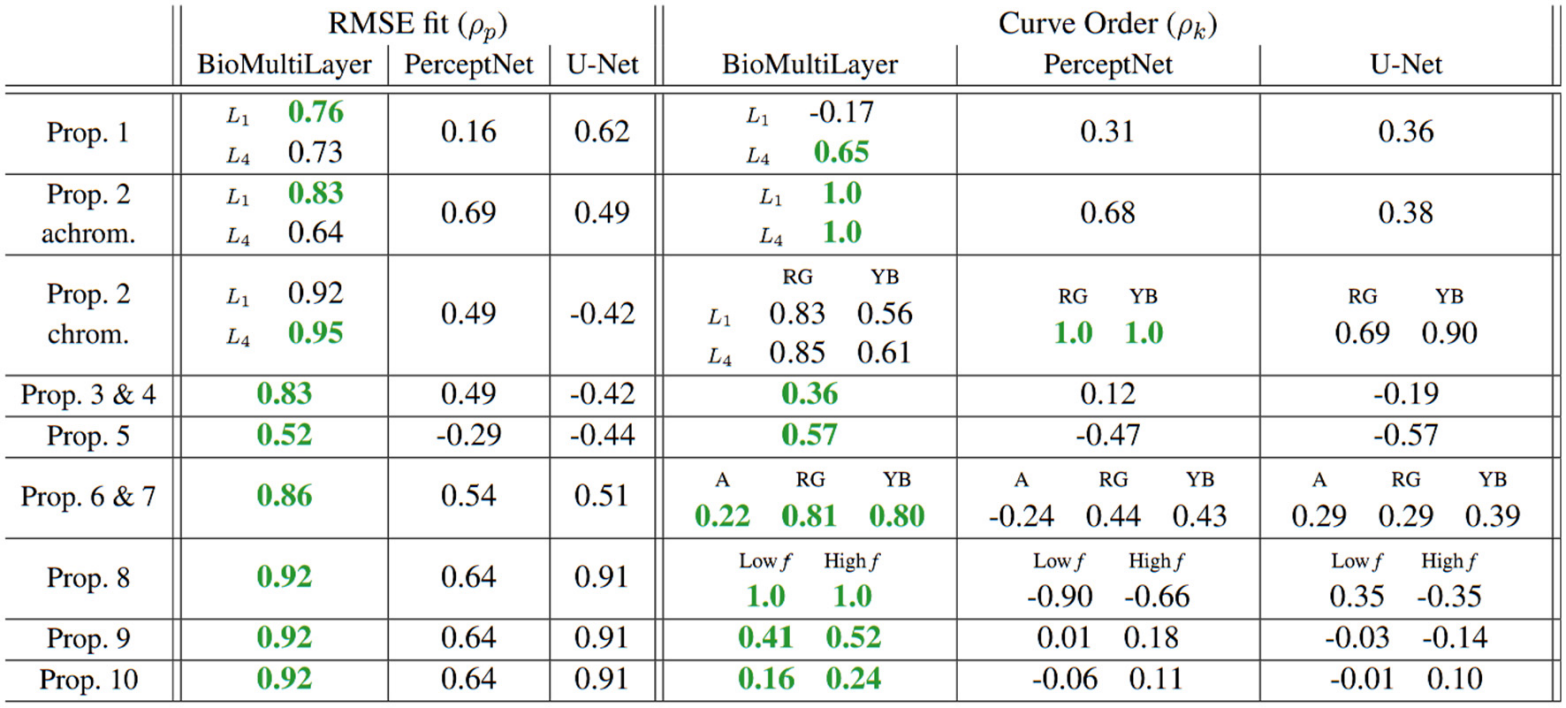
Alignment with human behavior measured in two ways: alignment between ground truth and prediction (Pearson correlation, *left panel*) and preservation of order between curves (rank Kendall correlation, *right panel*). Props. 8–10 share the same ρ_*p*_ values here because they share the same (known) no-masking curves. In props. 8–10, the interesting masking behavior is described by the order of the curves, quantified by ρ_*k*_.

The table in Figure 13 shows the quantitative description of the alignment with humans of each model for each property using the method proposed in Section 3.3 that considers RMSE alignment up to a scale factor using Pearson correlation and preservation of the order between curves using a rank correlation (in this case, Kendall correlation). All the scores are in a comparable [−1, 1] range, where bigger value means higher alignment. The table shows separate descriptors for read-outs done at different layers (*L*_1_ or *L*_4_), for the order of the curves for properties measured at different chromatic channels (*achromatic, red–green, and yellow–blue*), or the order of the curves for different frequencies (*Low f* and *High f* ). Maximum correlations for each property are highlighted in green.

The plain average of the descriptors, as suggested in BrainScore (Schrimpf et al., 2018), leads to the following model ranking: ***BioMultiLayer*** : **0.68**±0.14, ***PerceptNet*** : **0.26**±0.26. ***Bio-UNet*** : **0.20**±0.34, and Two-sample Kolmogorov-Smirnov tests (Monahan, 2011) show that the *BioMultiLayer* is significantly more aligned with humans than the other two (with *p* = 8·10^−3^ and *p* = 7·10^−3^, respectively), while the other two models are not significantly different (*p* = 0.26). This global conclusion is consistent with the qualitative analysis shown above.

The trend of individual scores in the table of quantitative descriptors agrees with the qualitative analysis shown in Table 2. Nevertheless, as anticipated in Section 3.3, the numerical descriptors may miss certain qualitative differences. For example, the rank correlation for the chromatic case of Property 2 in *PerceptNet* states that the alignment is good for both the RG and the YB cases. However, inspection of plots P and Q in Figure 10, compared with the expected qualitative behavior in Figure 6, shows that while the order in the RG curves of plot (Figure 10). P is correct, the shape of the curves is clearly not sigmoidal (i.e., wrong).

On the other hand, plain aggregation of correlation results over many (arbitrarily selected) phenomena, as done here following BrainScore (Schrimpf et al., 2018), has an obvious impact on the power of the descriptor. Bootstrap experiments on the results of the quantitative table illustrate this fact in Supplementary material.

In summary, the goal of the proposed “visual” Turing Tests is to allow one to “experience” the shape of the response curves by looking at the test stimuli generated by the software; however, the suggested quantification also allows a blind assessment of the alignment between networks and humans. Nevertheless, like any cost function, the value of the specific aggregated quantitative descriptor has to be taken with caution, and it is essential to always check that it properly captures the relevant qualitative (visual) behavior.

## 5 Discussion: what can be learned from the proposed methodology?

In this section, we discuss the benefits of the proposed *Decalogue* for generic artificial models. Benefits go beyond the evaluation of the human nature of models: even if we don't need a certain model to be similar to humans, the behaviors described by the human-like curves elicited by the stimuli in the *Decalogue* imply human-like bottlenecks and adaptation properties that one would like in efficient and robust artificial vision systems. Similarly, we also discuss the benefits of the architectures from classical vision science models that reproduce such behaviors.

### 5.1 (Non-human) curves suggest changes in the models

Failures to achieve the expected result in the proposed Test may suggest changes in the parameters of the models or even in their architecture. This is easy to see in the models considered here because they are relatively simple and interpretable, but this may also be the case in more recent (more complex) models.

**Figure 14:**
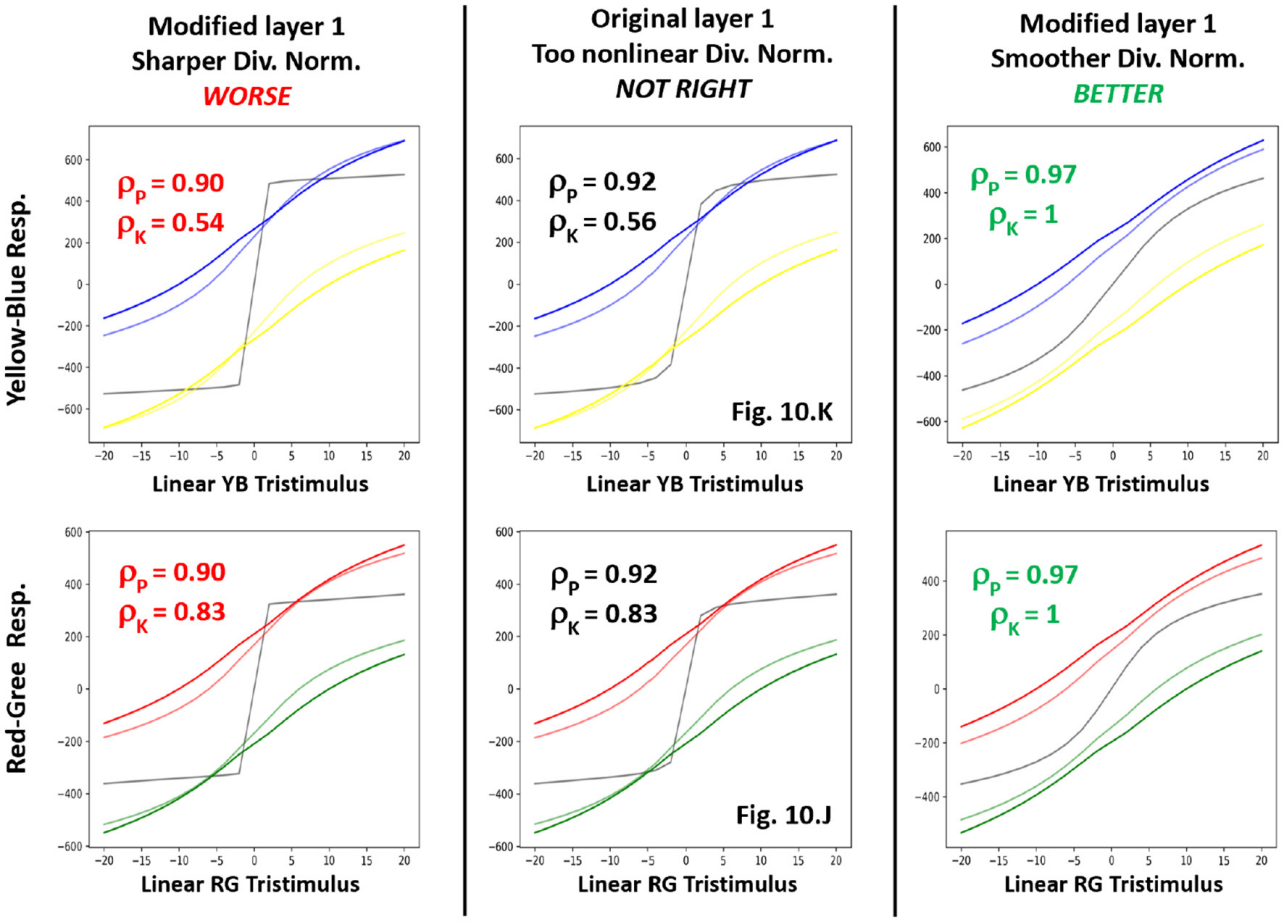
Fixing errors in a model (BioMultiLayer) from the results of the test. *Central Column*: displays the non-linear YB and RG responses of the first layer of the original BioMultiLayer (as depicted in Figures 10J, K). **(Left)** and **(Right)** panels show the effect of different interventions in the parameters of the model (see text).

First, consider, for instance, the non-human (not-right) behavior of the *BioMultiLayer* in the perception of color in the central panel of Figure 14. In that case, it displays too non-linear (too-sharp) responses to YB and RG stimuli in the no-adaptation case (curves in gray). As this model is made of interpretable divisive normalization layers^11^, the strength of the non-linearity can be modulated by the relative weight of the pool in the denominator. Following that intuition, we made two modifications to the original configuration of the first layer of the *BioMultiLayer*: we decreased and increased the parameter *b*_*i*_ that controls the relative weight of the pool in the denominator. By doing so, and re-computing the responses to the color stimuli in the test) we get the behaviors shown in the left and right panels of Figure 14: the interventions either alleviate the problem (right panel) or make it worse (left panel). This can be seen both in the qualitative shape of the curves and in the quantitative scores. The same rationale can be applied to other failures (e.g., too sharp/smooth responses in Figure 12, subplot P for textures) as suggested in Martinez et al. (2019).

**Figure 15:**
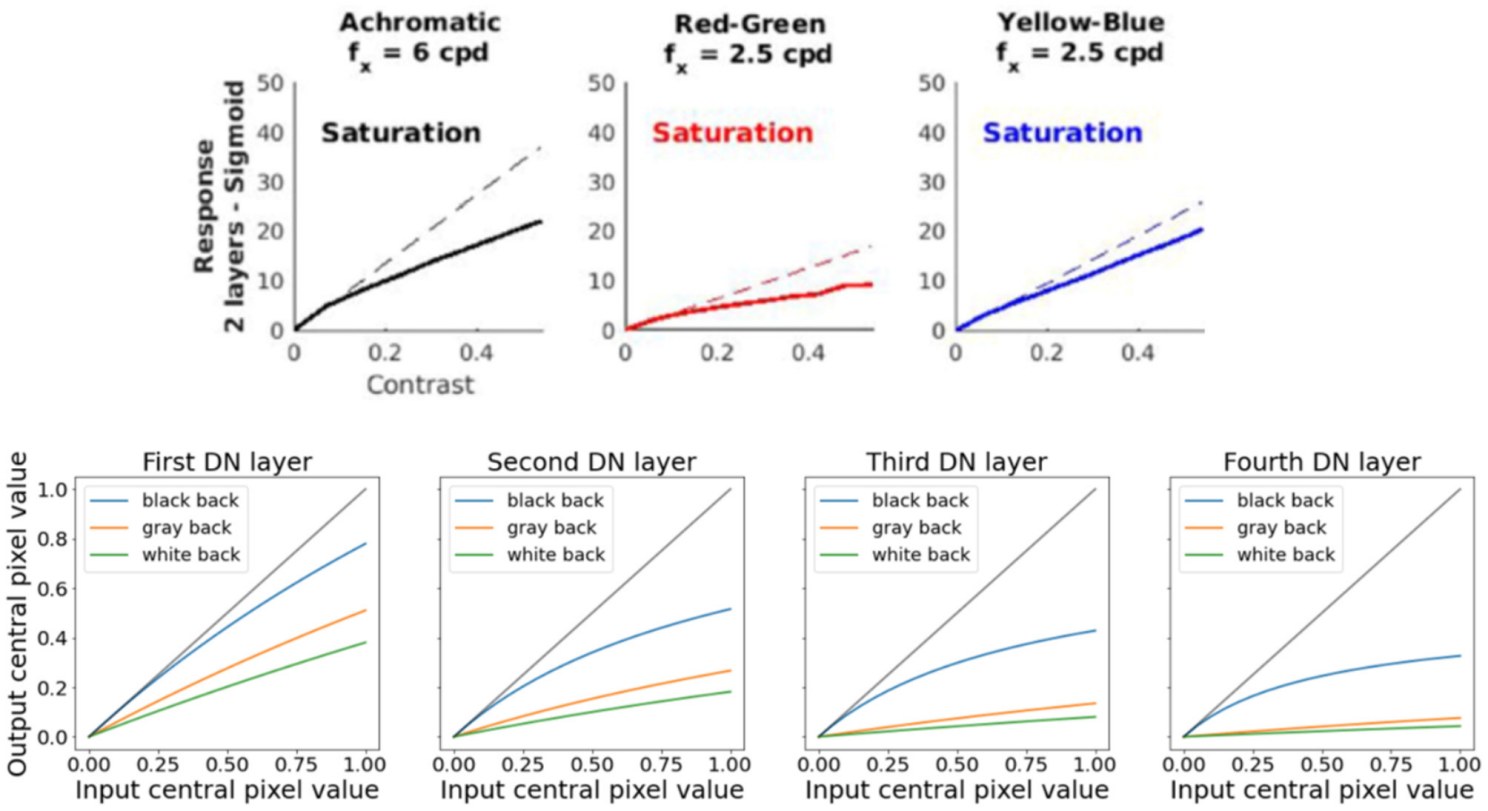
**(Top)** Human-like saturation behavior in contrast response (Property 6) happening in generic shallow autoencoders (Li et al., 2022). **(Bottom)** Human-like behavior obtained in image segmentation U-Nets when they are equipped with bio-inspired divisive normalization to improve their performance (Hernández-Cámara et al., 2025b).

When measuring the response of conventional networks using the spatially and chromatically calibrated stimuli proposed here, one can get human-like behaviors such as the ones shown in Section 3. For instance, shallow autoencoders optimized for image deblurring and denoising display human-like saturation when responding to achromatic and chromatic gratings of controlled spatial frequency: see Figure 15 (top), reproduced from Li et al. (2022). In this case, the slope of the response of these autoencoders (their sensitivity) is bigger for achromatic gratings than for red-green and yellow-blue gratings (Properties 3 and 4), and it reduces with the contrast of the gratings, just as in humans (Property 6).

As shown in the example above, this adaptive behavior and its benefits can be enforced in conventional networks by changing their architecture, for instance, by including divisive normalization or alternative bio-inspired non-linear layers. Examples include benefits in autoencoding and compression (Malo et al., 2006; Ballé et al., 2017), denoising and enhancement (Gutiérrez et al., 2006; Laparra et al., 2017), segmentation (Hernández-Cámara et al., 2023; Hernández-Cámara et al., 2025b), classification (Coen-Cagli and Schwartz, 2013; Bertalmío et al., 2020; Miller et al., 2022), or robustness to adversarial attacks with few layers given the strong non-linearity due to this kind of biological computation (Bertalmío et al., 2020). Moreover, the inclusion of these non-linearities, if done parametrically, (e.g., by using parametric expressions) reduces the training time and increases generalization because of the drastic reduction in the number of parameters of the network (Vila-Tomás et al., 2025).

Of course, for more complex (non-interpretable) models, the interventions may not be that simple. However, enforcing the behaviors shown here, for instance by imposing certain band-pass behaviors (similar to properties 3 and 4) through regularization, will change the models and may improve their results. For example, complex vision-language models with human-like CSFs also have human-like responses to some adversarial attacks (Hernández-Cámara et al., 2025a). Testing vision-language models with the stimuli in the proposed test is particularly interesting because one can interact with the model verbally (as done in humans) and ask if there are visible patterns in the images. In that way, sensitivities can be derived via psychometric functions (Hernández-Cámara et al., 2025a) as opposed to using (arguable) distortion metrics in the internal representations of the model [as done, for instance, in Li et al. (2022) and Akbarinia et al. (2023) and illustrated in Section 2 of Supplementary material].

### 5.2 Changes in the optimization goal or data statistics to achieve human-like adaptation

Misalignment with human behavior in proposed tests may also suggest changes in the optimization goal and in the statistics of the training data.

For instance, it is known that information maximization arguments lead to the emergence of Gabor-like receptive fields tuned to achromatic and opponent-chromatic directions (Hyvarinen et al., 2009; Gutmann et al., 2014). However, that sensible goal can be complemented with denoising-deblurring tasks so that center-surround cells and proper contrast sensitivity do emerge (Atick et al., 1992; Karklin and Simoncelli, 2011; Lindsey et al., 2019; Li et al., 2022). Moreover, if the contrast non-linearities do not emerge, they may be enforced by the segmentation goal in the encoder, as in (Hernández-Cámara et al. 2025b; see Figure 15, bottom), which shows curves where excitation is moderated by the presence of active neighbors. Regarding the poor emergence of plausible receptive fields in the considered non-parametric models (PerceptNet and BioUNet), this *error* makes sense in the context of the recently proposed *feature-spreading* problem (Vila-Tomás et al., 2025): if the goal is not demanding enough (as is usual in conventional goals), the features spread along all layers of the net in a way that the weak goal(s) is (are) fulfilled, but the layers remain biologically non-plausible.

**Figure 16:**
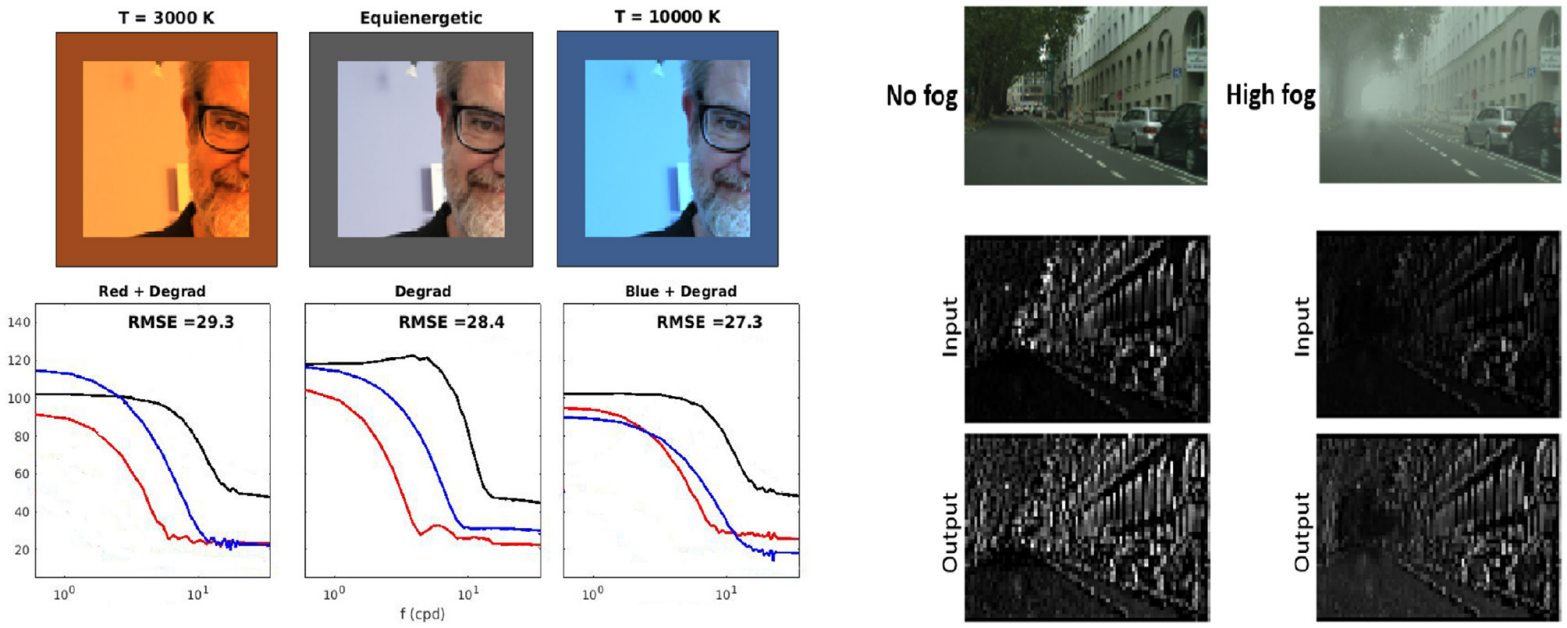
**(Left)** Adaptation in the CSFs in autoencoders obtained from training in the proper (colormetrically calibrated) environments for color adaptation (Li et al., 2022). **(Right)** Improved contrast perception by using bio-inspired divisive normalization (with adaptive contrast responses such as the ones described in our proposal) in the model (Hernández-Cámara et al., 2023). Images reproduced with permission from: M. Cordts, M. Omran, S. Ramos, T. Rehfeld, M. Enzweiler, R. Benenson, U. Franke, S. Roth, and B. Schiele, “The Cityscapes Dataset for Semantic Urban Scene Understanding,” in *Proc. of the IEEE Conference on Computer Vision and Pattern Recognition (CVPR)*, 2016.

Regarding suggestions on the training data, the behavior of the achromatic and chromatic CSFs proposed here was checked in Li et al. (2022) in scenes with well-controlled illumination. The behavior found in the autoencoder CSFs in those cases resembles Von Kries adaptation, as anticipated in (Gutmann et al. 2014). Figure 16 (left) shows that under low-temperature (reddish) illumination, the red-tuned channel is relatively attenuated with regard to the blue-tuned channel, and the other way around under high-temperature (bluish) illumination, as would happen using a Von Kries computation (Fairchild, 2013) or imposing the shifts in the response curves (Krauskopf and Gegenfurtner, 1992) shown in the Decalogue.

Finally, the emergence of the contrast-dependent non-linearities of property 8 (or the ability for contrast enhancement) may be enforced by including low-contrast images in the training of regular networks. For example, Figure 16 (right) shows that a network using Div. Norm. leads to contrast enhancement despite it not being trained on high-fog images, as anticipated by the contrast-dependent non-linearities shown in Figure 15 (bottom). However, in regular UNets (that do not include Div. Norm.), this behavior can be obtained (to a lesser degree) by including high-fog images in the training (Hernández-Cámara et al., 2023). In fact, the behavior in the BioUNet that includes Div. Norm. is not completely human (plot 12.N is ok but plots 12.O or 12.U are not). This may be in part due to a lack of constraints in the Div. Norm. [free kernels in Hernández-Cámara et al. (2023) and Hernández-Cámara et al. (2025b) as opposed to more sensible parametric kernels in Martinez et al. (2018, 2019)], but also because its behavior was obtained by training only with good-quality (clear day) images.

Of course, the limitations imposed by low-dynamic range and 8-bit quantized images imply that the artificial systems face scenes with limited variability, and hence, they may not develop certain non-linearities that are more pronounced (or conspicuous) in humans. In fact, including biologically inspired non-linear layers to cope with such variability (as suggested in Section 5.1) is an easy way to improve the efficiency of gamut mapping techniques (Laparra et al., 2017) and the robustness of networks devoted to vision (Hernández-Cámara et al., 2023; Hernández-Cámara et al., 2025b; Bertalmío et al., 2020).

### 5.3 Human-like curves imply better priors for natural image statistics

Two examples may illustrate how the non-linear responses to Gabor stimuli shown in textured contexts as presented in the proposed Decalogue capture the statistics of natural images: the described non-linear behaviors are a robust prior that may benefit anynetwork intended to work in vision.

**Figure 17:**
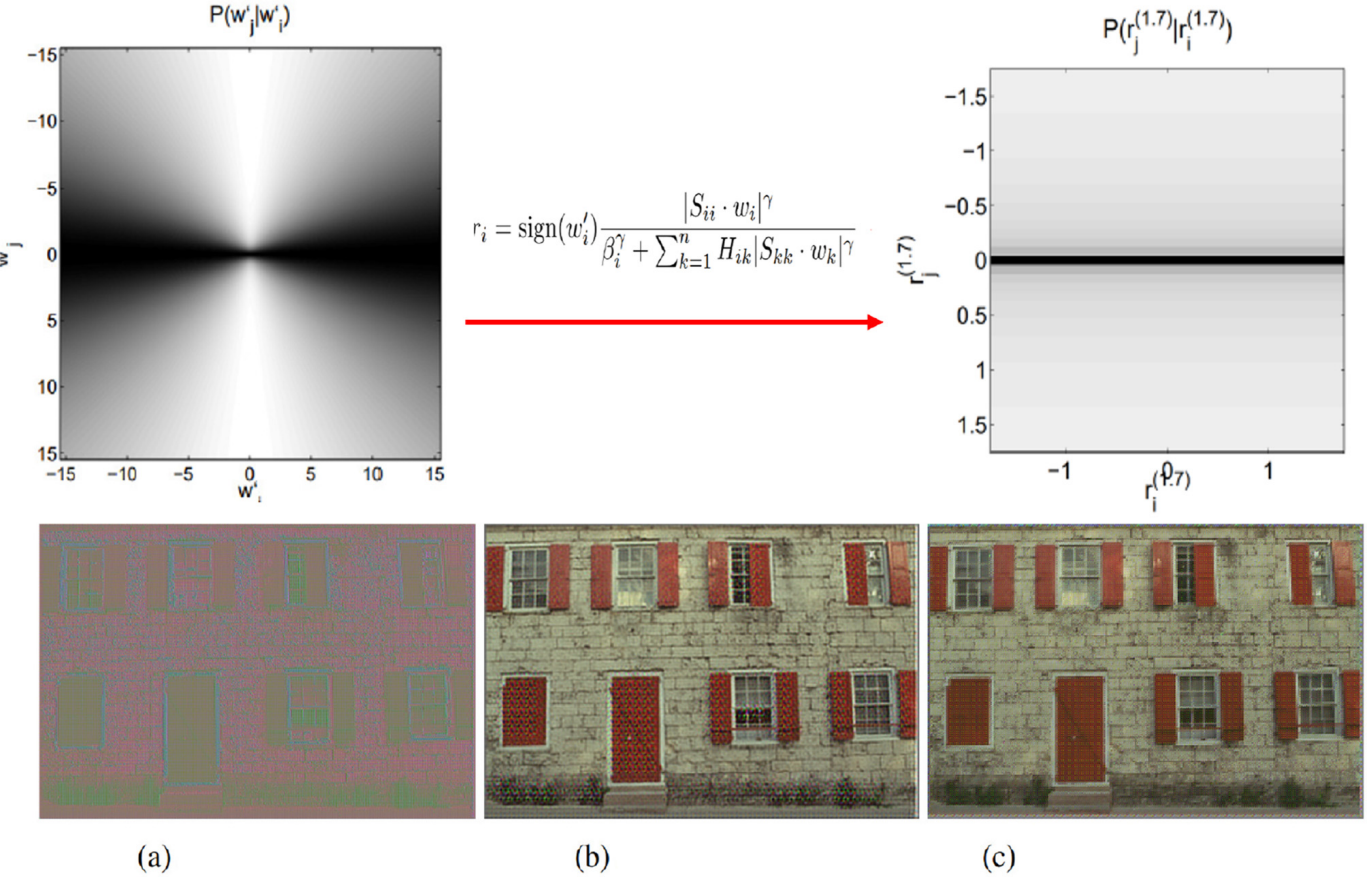
**(Top)** Image PDF factorization from the contrast non-linearites (div. norm.) illustrated in Figures 7, 8, as shown in Malo and Laparra (2010). **(Bottom)** autoencoders trained with distortion metrics based on the contrast non-linearities described in our proposal capture natural image statistics despite being trained with few samples (Hepburn et al., 2022).

First, in Figure 17 (top) we show that the energy of neighbor Gabor-like coefficients is correlated [bow-tie conditional probabilities of Gabor coefficients in natural images, as reported in Schwartz and Simoncelli (2001)], but the non-linear responses in textured backgrounds make the resulting coefficients independent (Malo and Laparra, 2010). In this regard, interactions between coefficients (e.g., as in Div. Norm.) are strictly required to remove redundancy because it is known that mutual information (redundancy) is invariant under point-wise transforms (Laparra et al., 2025).

Second, non-Euclidean metrics based on the non-linear responses to the stimuli presented here [e.g., metrics like those reported in (Laparra et al. 2010, 2017); Martinez et al. (2019); Malo et al. (2024)] represent a robust prior of the PDF of natural images as illustrated by the fact shown in Figure 17 (bottom): in autoencoders with access to very few samples, the use of this kind of perceptual metric in the loss function, makes the reconstruction of images much more robust than those using (naive) Euclidean metrics because the perceptual metric is already capturing the statistics of natural images, although samples are missing (Hepburn et al., 2022).

## 6 Final remarks

First, we noted that there are many open problems when we evaluate the human nature of artificial networks: there is a non-trivial relationship between the training environment, the task, and the architecture (Poggio, 2021; Malo and Hernández-Cámara, 2024; Hernández-Cámara et al., 2025c). That complexity implies it is difficult to choose the layer(s) to measure from and the read-out mechanism to check the human nature of the model responses. These problems point out the need for new tests of human alignment that are independent of the training data and goal.

This motivates our proposal: a set of stimuli, *a Decalogue*, based on classical low-level vision science. The stimuli and associated human responses describe the adaptive information bottleneck in the retina-V1 pathway. On the one hand, some of the sensitivity surfaces are standardized (Wyszecki and Stiles, 2000; Watson and Ramirez, 2000; Watson and Malo, 2002; Mullen, 1985; Daly, 1990; Malo et al., 1997; Kelly, 1979), or data is readily available (Blakemore and Campbell, 1969; Díez-Ajenjo et al., 2011) and allow quantitative comparison [namely properties 1, 3, 4, 5, 6], as done in Vila-Tomás et al. (2023), Li et al. (2022), Akbarinia et al. (2023), Hammou et al. (2025), and Cai et al. (2025). On the other hand, we showed that the responses involving tests in different illuminations or textured backgrounds (namely properties 2, 7–10) have clear qualitative trends that allow a quantitative assessment of the rank of the curves. As a consequence, we proposed a numerical description of the alignment combining Pearson and Kendall correlations.

This qualitative/quantitative analysis of the responses was applied to evaluate and rank three illustrative models: (1) a parametric one based on physiology, classical psychophysics, and maximum differentiation measurements (Martinez et al., 2018, 2019; Gomez-Villa et al., 2020a; Malo et al., 2024), (2) a non-parametric model, the *PerceptNet* (Hepburn et al., 2020), that includes trainable divisive normalization to reproduce human opinion on subjective image quality, and (3) a U-net with the same encoder as the *PerceptNet* but trained for image segmentation (Hernández-Cámara et al., 2023; Hernández-Cámara et al., 2025b). Experiments show that the proposed tests illustrate in easy-to-see ways the impact of the read-out location and strategy. Moreover, the quantitative and qualitative results are consistent and successfully rank the models according to their different origins: the two models with less alignment have been trained for tasks that are not enough to fully explain human behavior or are too flexible so that they easily develop non-human behavior.

Finally, in the discussion, we have seen that the proposed test can be useful to modify the architecture of the networks, both in their linear and non-linear parts. The test is useful to question the tasks or restrictions that are used in training (e.g., infoMax, noise, compression bottlenecks, classification, and segmentation). It is also useful to question the data used in the training, either in their generality or balance. Moreover, we discussed how the use of human behaviors represented by the data in the proposed test gives rise to priors related to the statistics of natural images.

In summary, we argue that the analysis of any kind of network, not only those that are specifically dedicated to modeling human vision, but any devoted to vision, can benefit, in great measure, from seeing how they respond to the proposed test.

## Data Availability

The datasets presented in this study can be found in online repositories. The names of the repository/repositories and accession number(s) can be found in the article/Supplementary material.

## Footnotes

2 All made online available here since 2022: http://isp.uv.es/docs/TuringTestVision.zip.

3 In the *talk* (Malo et al., 2022), we mentioned the humorous comment of Dr. Paninski at NYU back in 2001 after he carefully listened to the details of our brand new model: *yes, yes, that is nice, but the brain doesn't work like that, does it?*.

4 In the *talk* Malo et al. (2022), we guessed that, following that skepticism, Barlow questioned our preliminary work on the use of Principal Curves to explain color and texture non-linearities of human vision purely based on image data, back in 2004 (Malo et al., 2004): *yes, that is interesting, but the visual brain may not work like that*.

5 See the script StimuliColorNonLinearities.m.

6 See the script StimuliColorNonLinearities.m of this work which also uses the Toolbox Colorlab (Malo and Luque, 2002) for calibration.

7 See the script StimuliMaskEnergy.m which makes extensive use of the Toolbox Vistalab (Malo and Gutierrez, 2002).

8 See the script StimuliMaskOrient.m, which makes extensive use of the Toolbox Vistalab (Malo and Gutierrez, 2002).

9 The Decalogue Toolbox is available here: http://isp.uv.es/docs/TuringTestVision.zip.

10 Spectrally narrow Gaussians (5 nm width) of constant energy centered on different wavelengths along the visible spectrum on top of a low energy flat spectrum, as in Figure 4. In this way all the stimuli can be faithfully represented in digital values.

11 The divisive normalization transform (Carandini and Heeger, 1994, 2012) is a function *y* = *f*(*x*) with this generic sigmoidal form: $y_{i}=K_{i}\cdot \frac{\left|x_{i}\right|}{b_{i}+\underset{j}{\sum }H_{ij}|x_{j}|}$ in which each output *y*_*i*_ is driven by the corresponding input *x*_*i*_, *normalized* by a pool of the activity of the neighbor responses. It is a sort of local batch normalization (Krizhevsky et al., 2012a).
