# Supplementary material for: A Turing Test for artificial nets devoted to vision
Source: Front Artif Intell. 2026 Jan 5;8:1665874. doi: 10.3389/frai.2025.1665874 (PMC12812997; doi:10.3389/frai.2025.1665874)
Supplement: Supplementary file 1 [file Presentation_1.pdf]

frontiers

A Turing Test for Artificial Nets devoted to Vision (Supplementary Material)

Jorge Vila-Tomás [1†], Pablo Hernández-Cámara [1†], Qiang Li [2], Valero Laparra [1] and Jesús Malo [1,*]

[1] *Image Processing Lab, Universitat de València, Spain*
[2] *TReNDS, Georgia State, Georgia Tech, and Emory, GA, USA*
[†] *Equal contribution*

Correspondence*:
Corresponding Author
jesus.malo@uv.es

ABSTRACT

In this Supplementary Material we address the following issues: (1) we elaborate on the ground truth of the properties collected in the proposed Test. We describe the data or expressions to obtain the ground-truth curves and the routines of the associated software to obtain them. (2) We show an exhaustive example of the impact of the read-out location and read-out strategies in the considered models for a relevant characterization of low-level human vision: the achromatic and chromatic Contrast Sensitivity Functions. This points out that the proposed Test is useful to illustrate these open issues in model evaluation. (3) We carry out bootstrap experiments to show the impact of selecting a subset of properties of the proposed test on the aggregated score and analyze the discriminative power of such aggregate measure. This is a specific empirical illustration of the fact that results of aggregate scores have to be interpreted carefully.

Keywords: Evaluation of AI models, Neural Networks for Vision, Human Vision, Turing Test, Low-level Visual Psychophysics, Linear+Nonlinear cascade, Image Quality, Image Segmentation, PerceptNet

1 GROUND TRUTH FROM CLASSICAL EXPERIMENTS

All the ground-truth data can be computed from the Matlab/Octave routine `GroundTruthTuring.m` provided with the code associated to this work[1] which requires the use of the online available Toolboxes Colorlab [1] and Vistalab [2]. The data that is obtained from this function is shown in Fig. 1. Property 1 (Figs. 1.A and 1.B) corresponds to the V_λ and color matching functions of Jameson & Hurvich [3], and should be obtained by feeding the model under consideration with the stimuli generated by the function `StimuliSepectralSensitivities.m`. Property 2 (Figs. 1.C-E) correspond to the non-linear brightness response [4] (in dark background) and the nonlinear responses to linear saturation in the RG and YB directions (in gray background -no chromatic adaptation-) [5]. For the cases of different adaptation conditions one has to apply the qualitative trends shown in Figs. 5 and 6 of the main text,

[1] `http://isp.uv.es/docs/TuringTestVision.zip`

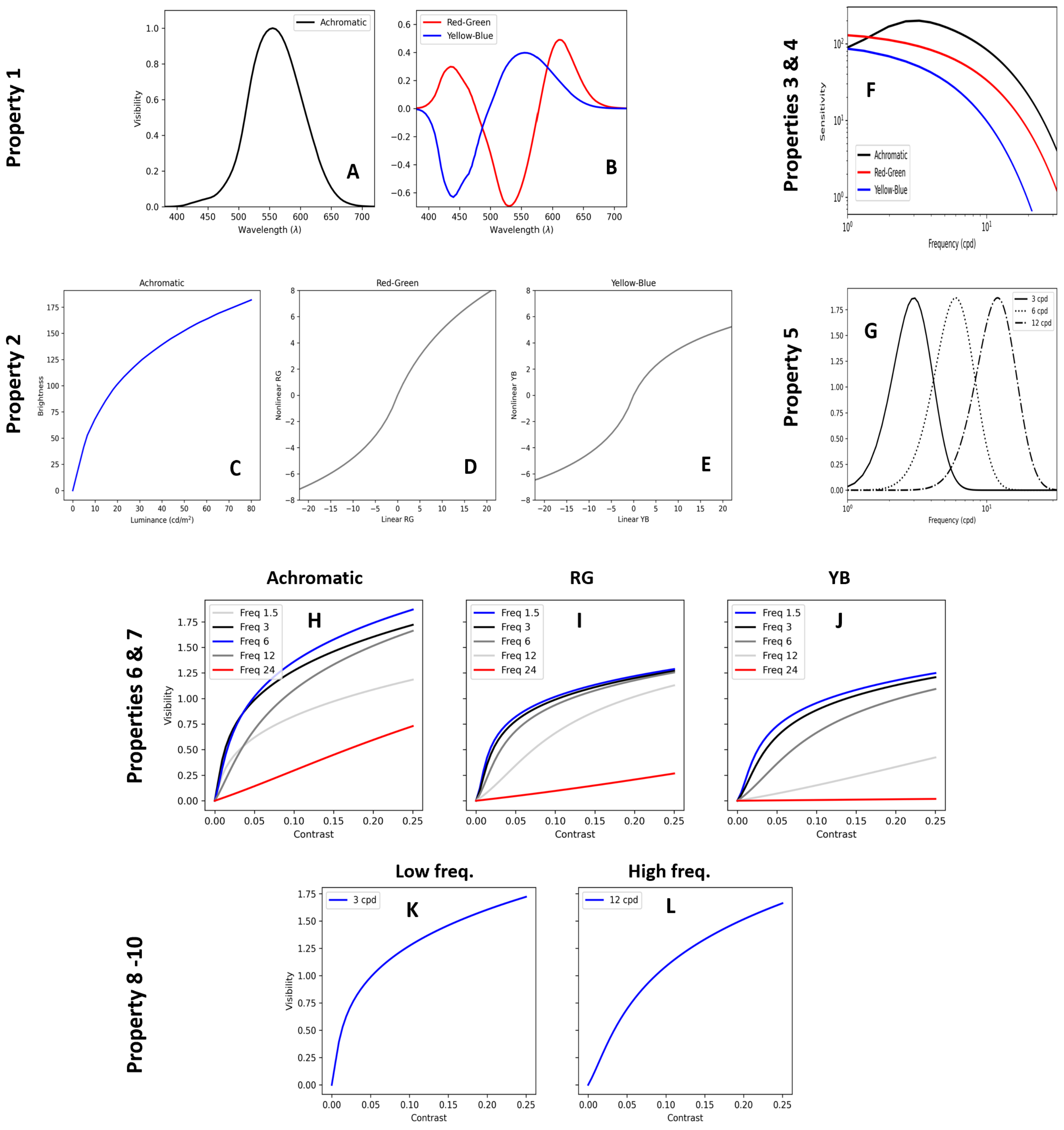

Figure 1. Ground truth curves of properties in the proposed Test that can be used for quantitative comparison using Pearson correlation ρ_p. In order to quantify the adaptive behaviors in Properties 2, and 8-10, one has to use the Kendall correlation ρ_k to measure the agreement with the rank of the curves that one would get according to the qualitative behavior described in Section 3 of the main text.

following the behavior reported in [6] and [5]. These curves should be obtained by models from the stimuli generated by `StimuliColorNonLinearities.m`. Properties 3 and 4 (Fig. 1.F) correspond to the classical achromatic and chromatic Contrast Sensitivity Functions (CSFs) [7, 8], following the

standardization of the Optical Society of America [9]. These curves should be obtained by models from the stimuli generated by the function `StimuliCSFs.m`. Property 5 (Fig. 1.G) corresponds to the frequency response of three wavelet-like receptive fields tuned to 3, 6 and 12 cpd fitted from adaptation of the CSFs following the classical Blakemore and Campbell experiment [10] through the function `blakemore_campbell_channel.m`. These curves should be obtained by models from the stimuli generated by the function `StimuliAdaptedCSFs.m`. Properties 6 and 7 (Figs. 1.H-J) correspond to the response to achromatic patterns of different frequency (Fig. 1.H) or chromatic patterns of different frequency (Figs. 1.I and H) of progressively increasing contrast. The response/visibility curves for achromatic patterns come from different fits of sensitivities based on the classical contrast matching data of Georgeson and Sullivan [11], or from measures of local sensitivity based on the inverse of contrast incremental thresholds [12]. Then, responses can be obtained by integrating the sensitivities along the contrast values as in [13, 14]. In particular, here we use the fit of Malo et al. [15], which is quite consistent with the fit of Daly [16] and the data of Georgeson and Sullivan [11]. Following the data gathered by Martinez-Uriegas at the Stanford Research International (SRI) institute, the suprathreshold behavior for RG and YB patterns is similar to the behavior for achromatic patterns up to a scaling given by the corresponding chromatic CSFs [17]. According to that, for those patterns we use the expression fitted by Malo et al. [15] plugging the corresponding chromatic CSFs of Mullen [8].

The sensitivity surfaces in the frequency-contrast plane obtained in that way can be computed with the function `thresholds_with_mask.m` and are shown in Fig. 2 for the achromatic and chromatic channels. The integration of these surfaces in the contrast dimension (following [13, 14]) for the corresponding frequencies leads to the curves in Figs. 1.H-J.

The curves in Figs. 1.H-J should be obtained by a model from the stimuli generated by the function `StimuliNoMaskATD.m`.

Properties 8-10 share the ground-truth curves for the considered frequencies obtained from the no-masking condition obtained from the Property 7 (shown in (Figs. 1.K and L)). Therefore, these curves should be obtained by a model also from the stimuli generated by the function `StimuliNoMaskATD.m`. However the key of properties 8-10 is masking for which there is not a general experimental source of data. Nevertheless, as described in Sections 3.2.3 and 3.2.4 of the main text, the fundamental effect of masking is

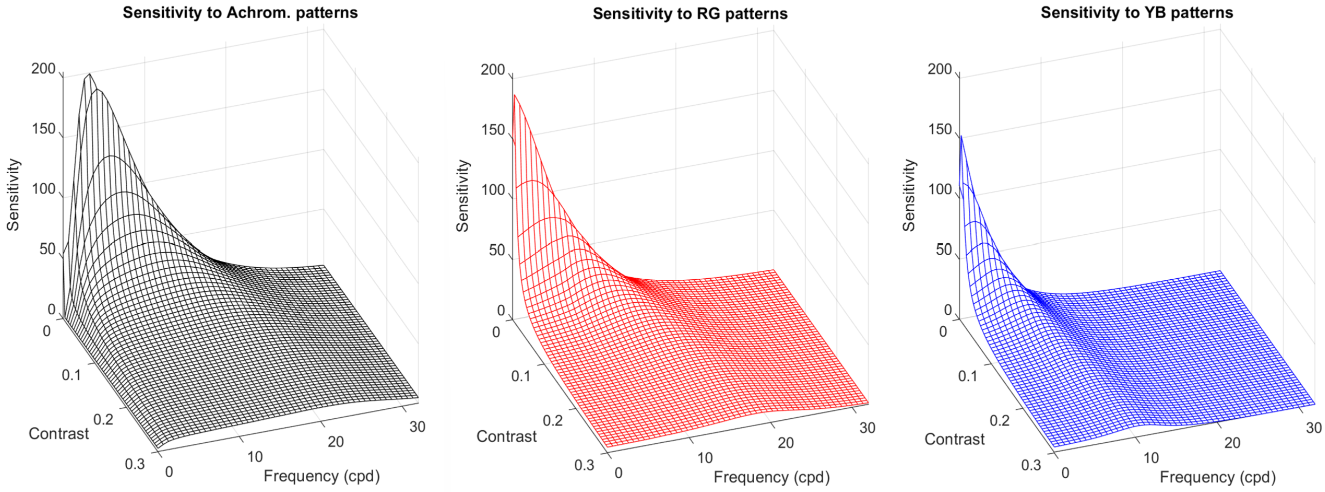

Figure 2. Sensitivity surfaces in the frequency-contrast plane of the Achromatic, Red-Green, and Yellow-Blue channels.

an attenuation that increases with the contrast of the background and the similarity between background and test, which allows to infer the proper rank in the masking conditions. The masking curves for properties 8-10 should be obtained by a model from the stimuli generated by the functions `StimuliMaskEnergy.m`, `StimuliMaskFreq.m`, and `StimuliMaskOrient.m`, respectively.

2 IMPACT OF READ-OUT LOCATION AND READ-OUT STRATEGY

Here we show that the proposed tests easily illustrate the point made in Section 2 of the main text about the impact of the read-out location and the read-out mechanism on the reproduction of human behavior. To do so, in this appendix we systematically study the impact of such factors on the achromatic and chromatic Contrast Sensitivity Functions (properties 3 and 4). We measured the CSFs at the four layers of the three considered models, using six different read-out strategies, namely five Minkowski summation norms and the cosine dissimilarity. On the one hand, one can define the visibility of patterns of frequency f as the difference between the responses of the networks at the selected layer, L, when facing a flat gray reference stimulus, $r_i^L(s_0)$, and the responses obtained for a low contrast stimulus of frequency f, $r_i^L(s_f)$, with Minkowski norm, q:

$$v_f = \left(\sum_i |r_i^L(s_f) - r_i^L(s_0)|^q \right)^{\frac{1}{q}} \tag{1}$$

We consider five summation norms: $q = 0.1, 1, 2, 4, 10$. These lead to read-out strategies with markedly different behavior: from *winner-takes-all* that favors large distortions in a small set of neurons ($q \gg$), to cases that favor the presence of small distortions in a large set of coefficients ($q \ll$). The considered norms also include the Euclidean measure ($q = 2$) used by default in the experiments of the main text. On the other hand, one can define the visibility of frequency f from the cosine dissimilarity between the response vector at layer L for the reference stimulus, s_0, and for the response vector for the stimulus of frequency f, s_f:

$$v_f = 1 - \frac{r^L(s_0)^\top \cdot r^L(s_f)}{|r^L(s_0)|\,|r^L(s_f)|} \tag{2}$$

Fig. 3 shows the achromatic CSFs along the four different layers of the three models obtained using the six considered read-out strategies. The classical inverted-U shape of the achromatic CSF [7] is only obtained at layers 3rd and 4th of the *BioMultiLayer* model and at the last layer of the *PerceptNet*. These results are interesting in terms of the interpretation of the layers. Note that the first layers (with almost-flat all-pass CSFs) do not have strong impact in the preservation or degradation of spatial texture, as expected from retinal (or prior to LGN) operations, focused on point-wise change of color representation. Then, the receptive fields derived from the adaptation of the CSF in the BioMultiLayer model (Fig.11.D in the main text) show that its last layer is functionally equivalent to V1 (which could be expected by its parametric wavelet-based construction). Note that this identification of layers based on their construction is different from the blind region-layer commitment used in BrainScore after the similarity scores have been computed [18]. Note, however, that the shape of the CSF is better reproduced by certain read-out strategies: in particular, the cosine similarity leads to narrower band-pass behavior while small Minkowski exponents ($q = 0.1$) lead to too-wide functions.

The quality of the curves along the depth of the models can be described by the quantitative scores defined in the main text and shown in Fig. 4. The aggregated score considers the Pearson correlation of the achromatic and chromatic CSFs (considered together to capture their relative scale) and the Kendall correlation to take into account the proper order of the curves. These quantitative results also reflect the

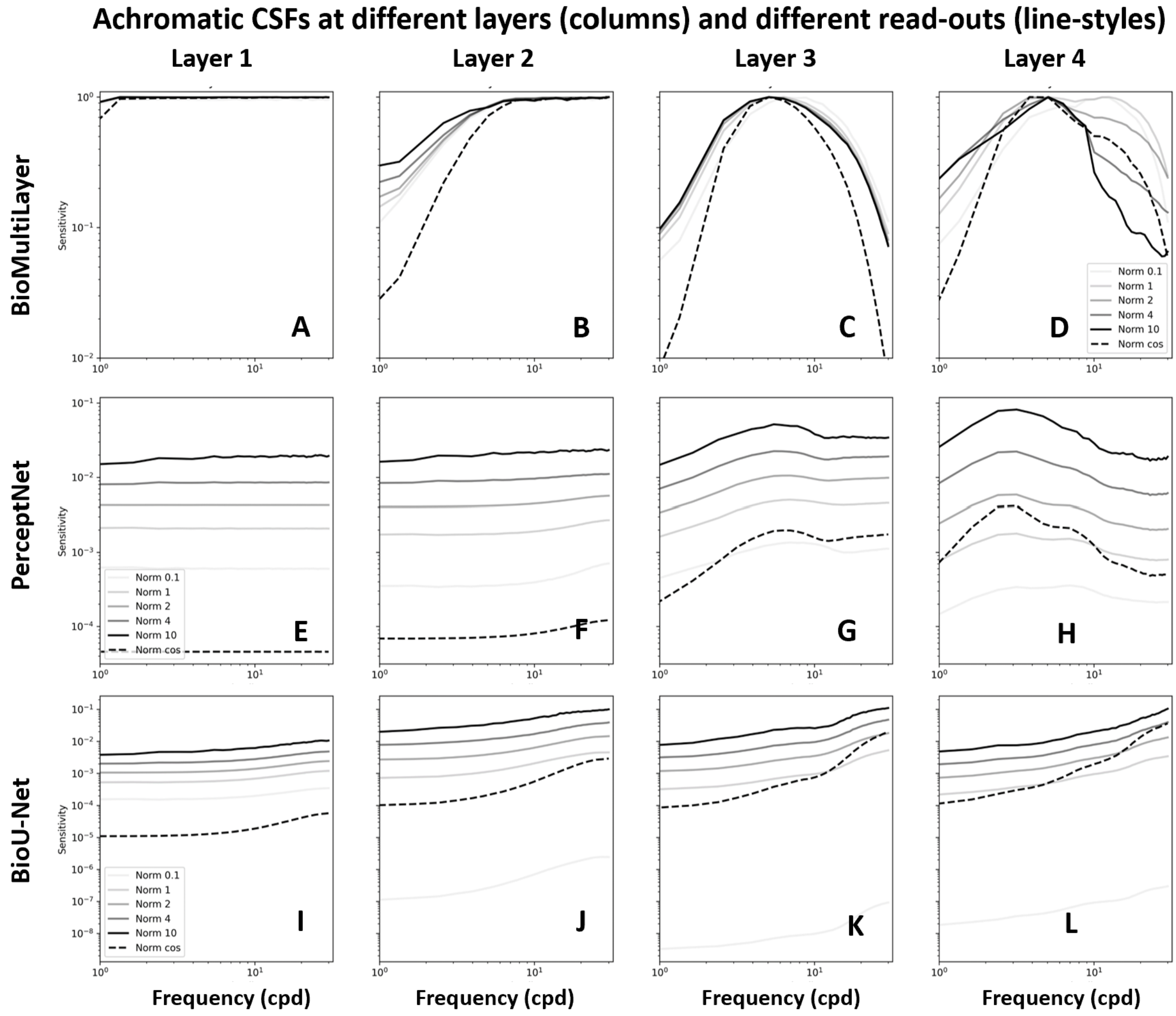

Figure 3. Achromatic CSFs of the three considered models at different layers computed with different read-out strategies.

higher alignment with humans of the *BioMultiLayer* model and the highly non-human behavior of the *BioUNet*. However, they also point out some of the limitations of the quantitative score. Note that, at the third layer of the *BioMultilayer* the score is very low for some of the read-outs because they introduce a wrong order between the RG and YB CSFs, thus lowering the score.

In summary, these exhaustive results for the CSFs illustrate the fact that ranking of models based on CSF alignment reported in [19, 20], which use different read-out strategies, may change using a different read-out choice.

3 EXPERIMENTS ON THE AGGREGATION OF NUMERICAL SCORES

Our selection of the properties to evaluate the alignment between models and humans is based on the characterization of the information bottleneck in low level vision (Section 3.1 of the main text). However, one may argue that this selection is arbitrary. Moreover, a summary of the alignment through a single aggregated numerical score is usually given for easy communication of the results [21]. Therefore, the specific ranking and the discrimination between models depends on the (somewhat arbitrary) selection of properties and the way individual scores are weighted when they are aggregated. As a result, these aggregated descriptions have to be handled with care. In this section we present experiments on the

t]

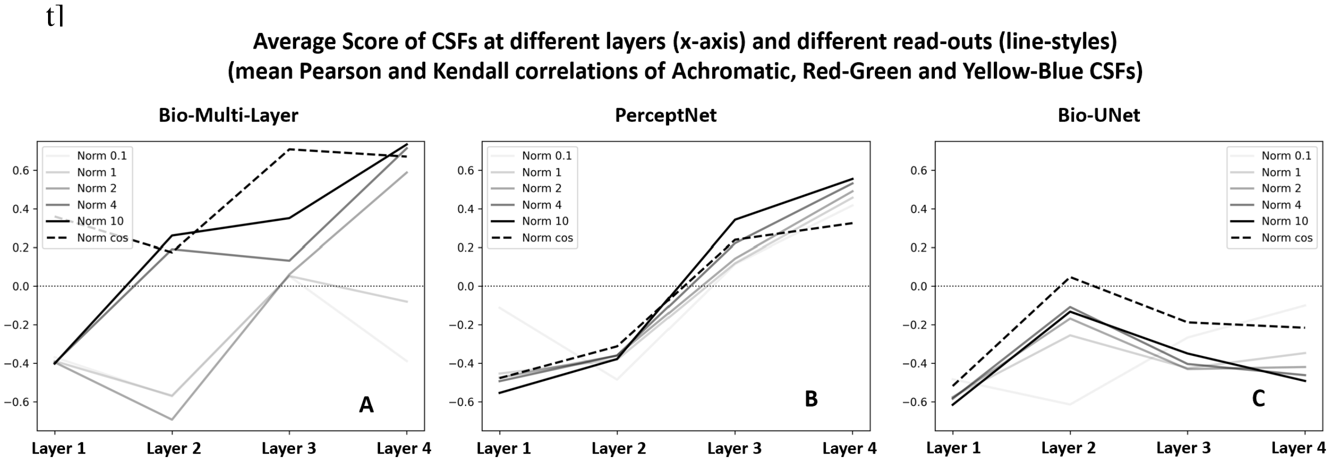

Figure 4. Quantitative scores for the Achromatic and Chromatic CSF at the different layers of the models computed from the CSFs obtained using different read-outs.

aggregation of the individual numerical scores to illustrate the impact of these decisions. In particular, we check the effect of selecting just a subset of properties and using different relevance weights for the Pearson and Kendall correlations.

If we randomly select $M < 10$ properties out of the 10 possible properties considered the proposed test (e.g. take M rows of Table 1, below) and average the correlations over the selected properties, we will have average values with corresponding deviations: $\rho_p \pm \sigma_p$ and $\rho_k \pm \sigma_k$. From these two descriptors, one can define a summary score, S, by aggregating the descriptors with a relevance weight, $w_p \in [0, 1]$: $S = w_p \rho_p + (1 - w_p)\rho_k$, and compute its uncertainty through standard error propagation: $\sigma_s = \sqrt{w_p^2 \sigma_p^2 + (1 - w_p)^2 \sigma_k^2}$. In the special case $M = 1$ (i.e. considering just one property) $\sigma_p = \sigma_k = 0$, but in that situation σ_s can be computed using the standard definition of weighted standard deviation over the two descriptors (ρ_p and ρ_k) for the considered property. One can tell if two models are significantly different according to such aggregated score with a non-parametric weighted two-samples Kolmogorov-Smirnov test [22] applied to the set of correlations for each model. In such weighted test one can establish different relevance factor for the Pearson and the Kendall scores (for instance because one may consider that ρ_p is a more reliable descriptor than ρ_k). The whole process (select a group of properties, compute the aggregated descriptors of the models with their corresponding deviation, and check if differences between models are significant) can be repeated for different randomly chosen groups of properties.

The results of 300 realizations[2] of such process are shown in Fig. 5. Each plot of the top row shows the average of the aggregated score obtained by the three models (in red, green and blue), taking random subsets of M properties, with a certain relevance weight, w_p, for the Pearson descriptors: *left* Pearson is considered to be less important than Kendall, *center* Pearson and Kendall are given equal relevance, and *right* Pearson is taken as more important than Kendall. The different colored lines in the plots at the bottom display the mean p-values obtained in the Kolmogorov-Smirnov tests comparing the descriptors from each pair of models. When the line is below the 0.05 level (dotted-line in black), the scores are significantly different, i.e. one model is significantly more aligned with humans than the other.

While the selection of a random subset of properties leads to a different aggregated descriptor, the consideration of all possible combinations (average over the 300 realizations of the evaluations) leads to

[2] Note that the number of combinations of M properties out of N properties is $\frac{N!}{(N-M)!M!}$, which, for $N = 10$ and $M \leq N$, has a maximum for $M = 5$, and is 252 combinations. Therefore, by taking 300 realizations we ensure we visit each combination at least once.

	RMSE fit (ρ_p)			Curve Order (ρ_k)		
	BioMultiLayer	PerceptNet	U-Net	BioMultiLayer	PerceptNet	U-Net
Prop. 1	**0.75**	0.16	0.62	0.24	0.31	**0.36**
Prop. 2	**0.84**	0.59	0.04	**0.86**	0.84	0.59
Prop. 3	**0.83**	0.49	-0.42	**0.36**	0.12	-0.19
Prop. 4	**0.83**	0.49	-0.42	**0.36**	0.12	-0.19
Prop. 5	**0.52**	-0.29	-0.44	**0.57**	-0.47	-0.57
Prop. 6	**0.86**	0.54	0.51	**0.61**	0.21	0.49
Prop. 7	**0.86**	0.54	0.51	**0.61**	0.21	0.49
Prop. 8	**0.92**	0.64	0.91	**1.00**	-0.78	0.00
Prop. 9	**0.92**	0.64	0.91	**0.47**	0.10	-0.09
Prop. 10	**0.92**	0.64	0.91	**0.20**	0.03	0.05

Table 1. Illustrative results to launch the experiments on score aggregation. In particular, this Table has been obtained from Table 3 of the main text by averaging the scores in the cells that contain fine grained values. However, note that slightly different values (e.g. by separating achromatic and chromatic results, or by selecting one of the represented layers or frequencies) do not change the trends, and conclusion, of the simulation below.

pretty stable average values for subsets of different size (horizontal lines in the top plots). However, if one takes small subsets of properties the differences in the scores in each realization are not statistically significant: the p-values are always above 0.05 for subsets smaller than 6 properties. On the other hand, considering large subsets of properties it can be safely said that the BioMultiLayer is more aligned with humans than the PerceptNet and the BioUNet. Giving different relevance to the descriptors (the different

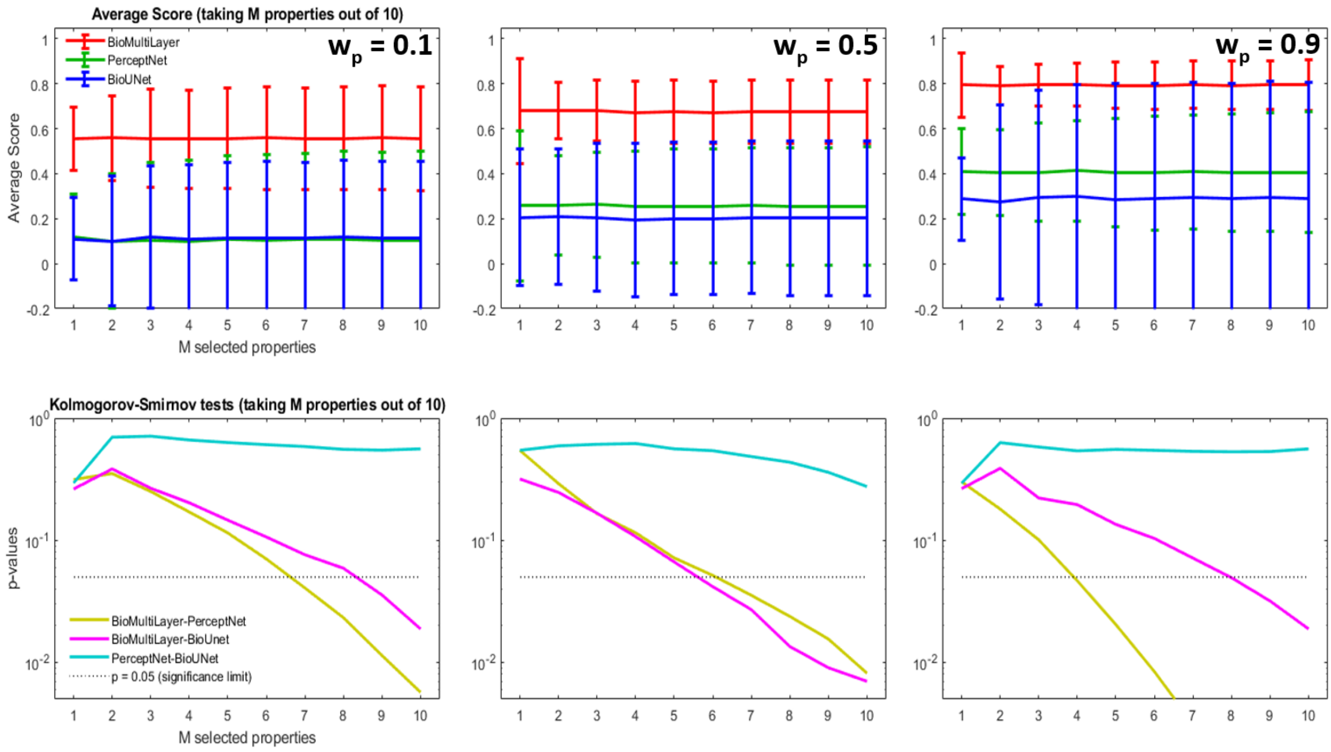

Figure 5. Effect of aggregation on the average score (top) and the discriminability between models (bottom) for randomly chosen subsets of M properties. Each column represents the results assuming different relevance weight w_p for the Pearson descriptor (the relevance of the Kendall descriptor is $1 - w_p$). The discriminability between models is represented by the p-values of a weighted Kolmogorov-Smirnov test with the correlations obtained by each pair of models.

columns) changes the values of the summary score: note the increase of the final score with the increase of the relative relevance of the Pearson descriptor. However, the p-values show that bigger differences in the final score do not necessarily imply larger discriminability.

The conclusion is that aggregated scores can be significant when a large number of properties is considered, but such summaries have to be interpreted carefully because they depend on the relevance given to the descriptors. Moreover, summaries cannot substitute fine grained analysis of the results on each property to understand the actual behavior of the models.

REFERENCES

[1] Malo J, Luque M. ColorLab: A Matlab Toolbox for Color Science and Calibrated Color Image Processing. *Univ. Valencia. https://isp.uv.es/code/vision_and_color/colorlab/content/* (2002).

[2] Malo J, Gutierrez J. VistaLab: The Matlab toolbox for linear spatio-temporal Vision Models. *Univ. Valencia. https://isp.uv.es/code/vision_and_color/colorlab/vistalab/* (2002).

[3] Hurvich LM, Jameson D. An opponent-process theory of color vision. *Psychological Review* **64, Part 1 6** (1957) 384–404.

[4] Wyszecki G, Stiles W. *Color Science: Concepts and Methods, Quantitative Data and Formulae* (New Jersey: John Wiley & Sons) (2000).

[5] Krauskopf J, Gegenfurtner K. Color discrimination and adaptation. *Vision Research* **32** (1992) 2165–2175.

[6] Whittle P. Brightness, discriminability and the "crispening effect". *Vision Research* **32** (1992) 1493–1507. doi:https://doi.org/10.1016/0042-6989(92)90205-W.

[7] Campbell F, Robson J. Application of Fourier analysis to the visibility of gratings. *Journal of Physiology* **197** (1968) 551–566.

[8] Mullen KT. The CSF of human colour vision to red-green and yellow-blue chromatic gratings. *J. Physiol.* **359** (1985) 381–400.

[9] Watson AB, Malo J. Video quality measures based on the standard spatial observer. *IEEE Proc. Int. Conf. Im. Proc.* (2002), vol. 3, III–III. doi:10.1109/ICIP.2002.1038898.

[10] Blakemore C, Campbell F. On the existence of neurons selectivity sensitive to the orientation and size of retinal images. *J. Physiol.* **203** (1969) 237–260.

[11] Georgeson M, Sullivan G. Contrast constancy: deblurring in human vision by spatial frequency channels. *J. Physiol.* **252** (1975) 627–656. doi:10.1113/jphysiol.1975.sp011162.

[12] Foley JM. Human luminance pattern-vision mechanisms: masking experiments require a new model. *J. Opt. Soc. Am. A* **11** (1994) 1710–1719.

[13] Watson A, Solomon J. A model of visual contrast gain control and pattern masking. *JOSA A* **14** (1997) 2379–2391.

[14] Shooner C, Mullen KT. Linking perceived to physical contrast: Comparing results from discrimination and difference-scaling experiments. *Journal of Vision* **22** (2022) 13–13. doi:10.1167/jov.22.1.13.

[15] Malo J, Pons A, Artigas J. Subjective image fidelity metric based on bit allocation of the human visual system in the DCT domain. *Image & Vision Computing* **15** (1997) 535–548.

[16] Daly S. Application of a noise-adaptive Contrast Sensitivity Function to image data compression. *Optical Engineering* **29** (1990) 977–987.

[17] Martinez-Uriegas E. Color detection and color contrast discrimination thresholds. *Proc. OSA Meeting* (1997), 81.

[18] [Dataset] Brain-Score Team. Developer clarifications — brain-score. `https://brain-score.readthedocs.io/en/latest/modules/developer_clarifications.html` (2024). Accessed: 2025-10-15.

[19] Li Q, Gomez-Villa A, Bertalmío M, Malo J. Contrast sensitivity functions in autoencoders. *Journal of Vision* **22** (2022). doi:10.1167/jov.22.6.8.

[20] Akbarinia A, Morgenstern Y, Gegenfurtner K. Contrast sensitivity function in deep networks. *Neural Networks* **164** (2023) 228–244.

[21] Schrimpf M, Kubilius J, Hong H, Majaj N, Rajalingham R, Issa E, et al. Brain-score: Which artificial neural network for object recognition is most brain-like? *bioRxiv* (2018). doi:10.1101/407007.

[22] Monahan J. *Numerical Methods of Statistics*. Cambridge Series in Statistical and Probabilistic Mathematics (Cambridge University Press) (2011).
